# Core Flipping in Lead Optimization: Rank Ordering Using λ‑Dynamics

**DOI:** 10.1021/acs.jcim.5c00320

**Published:** 2025-06-16

**Authors:** Parveen Gartan, Charles L. Brooks, Nathalie Reuter

**Affiliations:** † Department of Chemistry, 1658 University of Bergen, Bergen 5020, Norway; ‡ Computational Biology Unit, University of Bergen, Bergen 5020, Norway; § Department of Chemistry, 1259 University of Michigan, Ann Arbor, Michigan 48109, United States; ∥ Biophysics Program, University of Michigan, Ann Arbor, Michigan 48109, United States

## Abstract

In structure-based drug discovery, reliable structural models of ligands bound to their target receptors are critical for establishing the structure–activity relationship of the congeneric series. In such a series, substitutions on a common scaffold core might lead to different binding modes, ranging from slight changes of orientations to flipping or inversion of the core structure. Moreover, molecular docking might lead to alternative orientations within the top-ranked poses without being able to discriminate which is most likely. To determine the relative binding affinities between two alternative ligand poses, we propose a methodology based on relative binding free energy calculations using the λ-dynamics method. We used a dual-topology approach with distance-restraining schemes. We introduced a novel strategy using a one-step perturbation to calculate the contributions of the applied restraints. While using FEP/MBAR instead for that purpose led to smaller uncertainties, it suffered from convergence issues. We tested the validity and predictive power of our approach using two pharmaceutically relevant targets and eight small-molecule inhibitors from the experimentally characterized congeneric series. For each target, our approach correctly ranks the known X-ray poses as more favorable than alternative flipped poses. The proposed methodology can be easily extended to rank more than two poses and should also be applicable to the evaluation of alternative rotamers of target amino acids.

## Graphical abstract

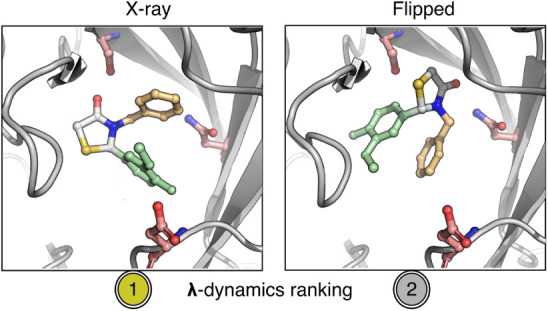

## 1 Introduction

The prediction of the structure or pose of a ligand bound to its target protein is a critical step in structure-based drug design (SBDD). It forms the basis for establishing the structure–activity relationship (SAR) of a series of small-molecule inhibitors, which is key information to enhance the activity of the compounds in both the hit-to-lead and lead optimization phases. In these early steps of drug discovery, the core structure of the compounds is typically decorated with different substituents with the aim to enhance binding affinity and optimize drug-like properties.^1^ Calculations of relative binding free energies (RBFE) have been increasingly used both in academia and industry to predict the relative affinities of a congeneric series of small-molecule inhibitors to a common target.^2–4^ The popularity of RBFE calculations is a consequence of the increased computational power available and the improvement of molecular mechanics force fields for small molecules,^5–9^ which have led to RBFE accuracies within 1 kcal/mol.^2–4,10^ Although, the accuracy of RBFE calculations is very sensitive to the pose of the ligand in the receptor.^11^

Given the sparsity of experimental structural data in the early stages of drug design campaigns, it is often assumed that different substitutions around a core structure lead to similar ligand binding modes. A single binding mode is, therefore, often used as a reference for the entirety of the congeneric series, including as a starting structure for RBFE calculations. However, on occasion, substitutions on the core might lead to a different binding mode, resulting from slight changes of orientations to flipping or inversion of the core structure.^12–18^ Moreover, molecular docking might lead to a variety of poses for a given ligand or within a congeneric series, and the top-ranked pose from docking might not always be alike the experimentally observed pose,^19–21^ because of the approximate conformational space exploration and empirical scoring functions used in docking methods.

Assuming the wrong ligand pose for a whole congeneric series, or for a subset of compounds, might jeopardize the reliability of the SAR derived from in vitro assays or the predictive power of the RBFE calculations. So, accurate computational methods able to distinguish between different poses are sorely needed.

A variety of exhaustive computational approaches have been developed to predict binding modes, focusing on improving the sampling of ligand binding poses using nonequilibrium candidate Monte Carlo (NCMC),^22^ sequential Monte Carlo,^23^ Hamiltonian replica exchange,^24^ or generalized replica exchange with solute tempering (gREST),^25^ to name a few. Simpler models or theories approximating the free energy of binding have also been used, such as Grid Inhomogeneous Solvation Theory (GIST)^18,26^ and linear interaction energy (LIE).^27^ Absolute binding free energy (ABFE) calculations can also be used to predict the more favorable binding mode when a priori information about the ligand binding mode is not available.^28–30^

Here, we propose taking advantage of the computational efficiency and high accuracy of RBFE calculations to identify the most likely pose when two or more alternatives are considered, such as from the top-scoring poses obtained from molecular docking. As stated above, RBFE calculations have become computationally affordable and accurate within 1 kcal/mol and present the advantage of not needing to sample large phase spaces. RBFE calculations that aim to predict the more favorable binding mode or pose when alternate poses or modes are considered require the use of a dual-topology model, where each pose is considered explicitly. In addition, these calculations require restraints to keep the noninteracting pose in the binding site of the target and avoid wandering off in the simulation box, which would result in convergence issues.^1,28,31–33^ The contribution of such restraints should be accounted for in a computationally efficient manner. Traditional RBFE calculation methods, i.e., free energy perturbation (FEP) or thermodynamic integration (TI), can only perturb one binding mode to another in a single calculation, thus potentially limiting the application (due to increased computational cost) of these calculations in the early phases of drug design where more than two poses might be considered (although this is not an issue in the present study). This bottleneck of RBFE calculations has been addressed by multistate methods^34–37^ that are more efficient than traditional windowing approaches. One such method is multisite λ dynamics (MSλD),^34^ an extension of the λ-dynamics method.^38,39^ λ-dynamics presents the advantage that the alchemical parameter λ is a dynamic variable, thus alleviating the need for the definition of intermediate states or windows. λ-dynamics has also been shown to efficiently sample different ligand orientations and conformations.^40^ MSλD has been shown to scale efficiently with an increasing number of perturbations and sites^4,41^ and is, therefore, a method worth exploring further in the context of distinguishing alternative binding poses. In our current approach, we use a dual-topology model, where each of the poses is an end-state in a single MSλD calculation (reducing to a λ-dynamics calculation). We also introduce the required restraints in the system to keep the ligand poses in the binding site when they are weakly coupled and to maintain the starting poses relative to the protein. Efficient sampling is achieved with the use of the adaptive landscape flattening algorithm (ALF),^42,43^ already available/implemented as part of the MSλD workflow in the CHARMM^44,45^ package. Our approach is described in detail in the Methods section. We show the applicability and validity of the proposed method on two actual drug targets for which the binding poses of congeneric ligands are known from X-ray data. For the purpose of clarity, since we are using sets of pairs of states, we refer to our simulations as λ-dynamics simulations or calculations. The method itself is still referred to by its standard name, i.e., multisite λ dynamics (MSλD), in the following text.

## 2 Methods

### 2.1 Thermodynamic Cycle for Flipped Pose Binding

We used the thermodynamic cycle shown in Figure 1. The horizontal processes refer to the free energy of binding of ligand L to protein P in its X-ray (top) and flipped (bottom) poses. We can alchemically transform the X-ray pose of ligand L to its flipped pose via the vertical legs of the cycle, each pose being considered a different substituent/end-state for an RBFE calculation. The relative binding free energy difference between the two poses can be obtained in eq 1. The vertical leg of the cycle corresponding to the relative solvation energy (left) of both poses cancels out, and hence only a single simulation is required to complete the cycle (right leg, $\Delta G_{L_{xray}\to L_{flip}}^{complex}$ ). This system can be represented by using a dual-topology model with the MSλD methodology, where all atoms of both poses are explicitly represented. The introduction of restraints in our RBFE calculations is needed to avoid the weakly coupled poses from wandering off in the simulation box, which can lead to convergence issues.^28,33^

**Figure 1:**
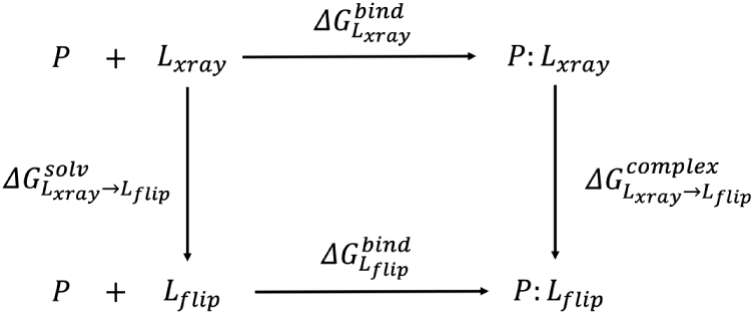
Thermodynamic cycle for calculations of the relative binding free energy of the flipped ligand pose to the protein compared to the X-ray ligand pose.


1 $$\Delta \Delta G_{xray\to flip}=\Delta G_{L_{xray}\to L_{flip}}^{complex}-\Delta G_{L_{xray}\to L_{flip}}^{solv}$$

2 $$\Delta \Delta G_{xray\to flip}=\Delta G_{L_{xray}\to L_{flip}}^{complex}$$

3 $$\Delta G_{L_{xray}\to L_{flip}}^{complex}=\Delta G_{xray\to flip}^{MS\lambda D}=-k_{B}T\ln\left(\frac{P_{\lambda_{flip}}=1}{P_{\lambda_{xray}}=1}\right)$$

### 2.2 Restraining Schemes

We used two different types of restraints depending on the system: either (a) ligand poses were restrained to one another or (b) ligand poses were restrained to the protein. For the first type of restraints, the pairs of atoms were chosen via visual inspection, and their contribution to the final free energy cancels out between the poses. The second type of restraint requires careful consideration, since it has the potential to introduce numerical instabilities or convergence issues in the system. We chose the multiple-distance restraining scheme introduced by Clark et al.^46^ as it is easily implemented, computationally efficient, and devoid of convergence or sampling issues. The restraints were applied between the protein and ligand poses during the λ-dynamics simulations.

The free energy difference corresponding to the binding of the flipped pose relative to the X-ray pose from λ-dynamics simulations with restraints can be written as in eqs 4 and 5, depending on the type of restraints used.

4 $$\Delta G_{xray^{\#}\to flip^{\#}}^{MS\lambda D}=-k_{B}T\ln\left(\frac{P_{\lambda_{flip^{\#}}}=1}{P_{\lambda_{xray^{\#}}}=1}\right)$$

where ^#^ indicates that the ligand poses are restrained to each other.

5 $$\Delta G_{xray^{**}\to flip^{**}}^{MS\lambda D}=-k_{B}T\ln\left(\frac{P_{\lambda_{flip^{**}}}=1}{P_{\lambda_{xray^{**}}}=1}\right)$$

where, ^∗∗^ indicates that the ligand poses are restrained to the protein using multiple distance restraints.

The restraining scheme by Clark et al. was proposed for use in ABFE calculations, and an analytical correction was used to account for its contribution.^46^ However, in our system, since the ligands are not annihilated, an efficient endpoint correction was applied to get their contribution, as described in the next section.

### 2.3 Contribution of Protein–Ligand Restraints with One-Step Perturbation

In principle, the contribution of the protein–ligand restraints can be obtained using windowing methods such as FEP or TI as endpoint corrections. However, FEP or TI would increase the computational cost of the workflow. We used the one-step perturbation (OSP)^47–49^ method instead, as it requires the simulation of a single reference state. The free energy of adding the restraints on the X-ray ( $\Delta G_{xray\to xray^{**}}^{OSP}$ ) and flipped poses ( $\Delta G_{flip^{**}\to flip}^{OSP}$ ) was obtained using the thermodynamic cycles shown in Figure 2A,B, respectively. The reference state is defined by assigning λ = 0.5 for both the X-ray and the flipped poses in the protein–ligand complex; both poses still have multiple distance restraints to the protein with the full value of the force constant.

**Figure 2:**
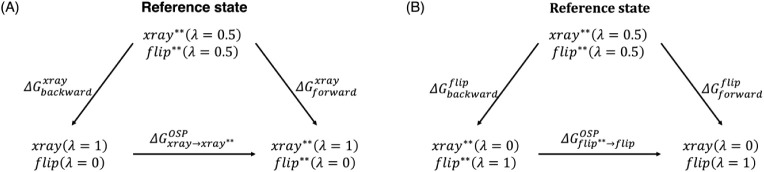
Thermodynamic cycle for evaluating the cost of (A) adding multiple distances to the X-ray pose and (B) removing multiple distance restraints on the flipped pose using the one step perturbation method. The reference state contains both ligand poses at λ = 0.5. ^∗∗^ represents the full NOE force constant.

The unidirectional free energies for transitioning from the reference state to the end-state corresponding to the X-ray pose without any restraints and the X-ray pose with multiple distance restraints (Figure 2A) were obtained using Zwanzig’s perturbation equation (eqs 6 and 7).

6 $$\Delta G_{backward}^{xray}=-k_{B}T\ln\langle \exp{[\frac{-\left(U_{target}-U_{reference}\right)}{k_{B}T}]\rangle }_{\mathrm{reference}}$$

where the target corresponds to the end-state in Figure 2A with an X-ray (λ = 1) and flip (λ = 0).

7 $$\Delta G_{forward}^{xray}=-k_{B}T\ln\langle \exp{[\frac{-\left(U_{target}-U_{reference}\right)}{k_{B}T}]\rangle }_{\mathrm{reference}}$$

where the target corresponds to the end-state (Figure 2A) with X-ray^∗∗^ (λ = 1) and flip^∗∗^ (λ = 0). The free energy of adding restraints to the X-ray pose, $\Delta G_{{xray\to xray}^{**}}^{OSP}$ is calculated as

8 $$\Delta G_{xray\to xray^{**}}^{OSP}=\Delta G_{forward}^{xray}-\Delta G_{backward}^{xray}$$

Similarly, the free energy of removing restraints from the flipped pose $\Delta G_{flip^{**}\to flip}^{OSP}$ is calculated as

9 $$\Delta G_{flip^{**}\to flip}^{OSP}=\Delta G_{forward}^{flip}-\Delta G_{backward}^{flip}$$

The free energies corresponding to the addition of restraints on the X-ray pose and removal of the restraints from the flipped pose were then combined with the free energy from MSλD to obtain the final relative free energy difference between the X-ray pose and the flipped pose (eq 10, Figure 3):

10 $$\Delta \Delta G_{xray\to flip}=\Delta G_{xray\to xray^{**}}^{OSP}+\Delta G_{xray^{**}\to flip^{**}}^{MS\lambda D}+\Delta G_{flip^{**}\to flip}^{OSP}$$

**Figure 3:**
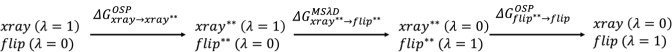
Terms of eq 10 for the calculation of the free energy difference between the flipped and X-ray poses from MSλD with multiple distance restraints ( $\Delta \Delta G_{xray\to flip}$ ).

## 3 Systems

We tested our approach on two drug targets: the human neutrophil elastase (HNE) and the *N*-myristoyltransferase (*Lm*NMT) and their respective ligands in congeneric series. These targets were chosen because of the availability of experimental structural data and because they represent different types of binding sites; HNE has a well-defined ligand binding site, while *Lm*NMT has a shallow and large binding site, rendering pose prediction particularly challenging. For each of these, X-ray structures of target–ligand complexes are available.

### 3.1 Human Neutrophil Elastase

HNE is a target for chronic lung inflammation. The latest generation of dihydropyrimidinone noncovalent HNE inhibitors (Figure 4) from Bayer HealthCare AG^50,51^ bind via shape complementarity to the enzyme. HNE has two small, well-defined binding pockets. HNE was also chosen because we previously reported good to excellent agreement between experimentally determined activity for 11 inhibitors and affinities predicted using MSλD with different force fields.^52^ The X-ray structures of all five small-molecule inhibitors with the enzyme show the same pose, with the trifluoromethyl phenyl ring binding to the so-called S1 pocket and the cyanophenyl ring in the S2 pocket (Figure 5A). Molecular docking predicts alternative poses for three of the five ligands in the series (see the Results section).

**Figure 4:**
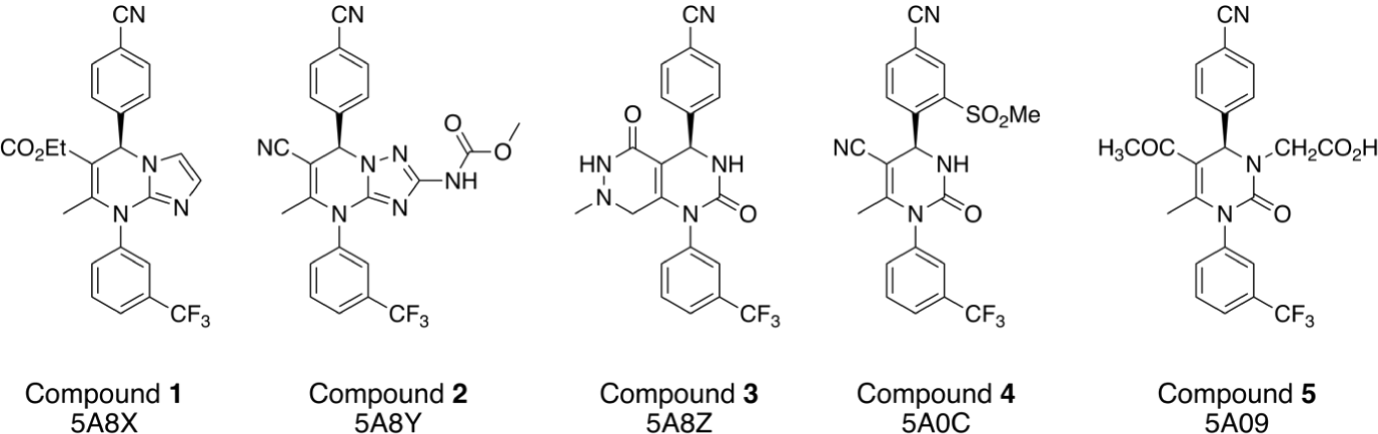
Selected dihydropyrimidinone noncovalent HNE inhibitors and the PDB IDs^50,51^ corresponding to their X-ray structures with HNE.

**Figure 5:**
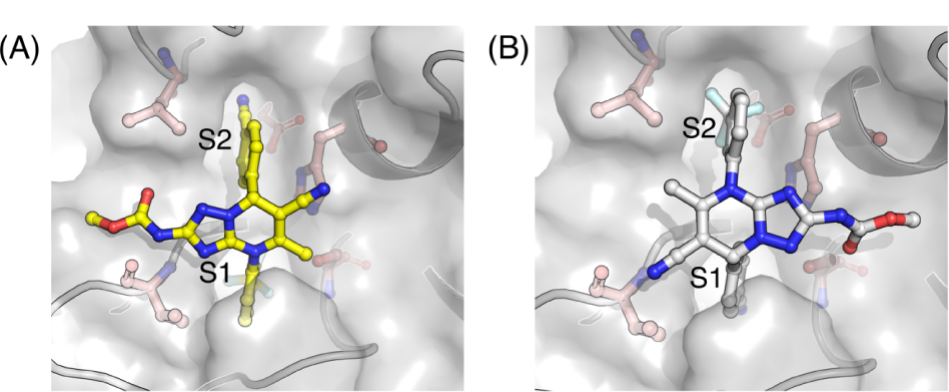
Compound 2 bound to HNE in the (A) X-ray pose and (B) flipped pose.

### 3.2 *N*-Myristoyltransferase

*Lm*NMT is a target for the development of therapies for the treatment of sleeping sickness (also known as human African trypanosomiasis, HAT). It has a fairly large and shallow pocket that binds small-molecule inhibitors. X-ray structures of the three thiazolidinone inhibitors (Figure 6) with the enzyme revealed two distinct binding modes (Figure 7A–C) where the thiazolidinone core is flipped between compound 2 on one hand and compounds 1 and 3 on the other.^53^ In what follows, we define the *flipped* poses of compounds 1 and 3 as the X-ray pose observed for compound 2, and vice versa (Figure 7D–F).

**Figure 6:**
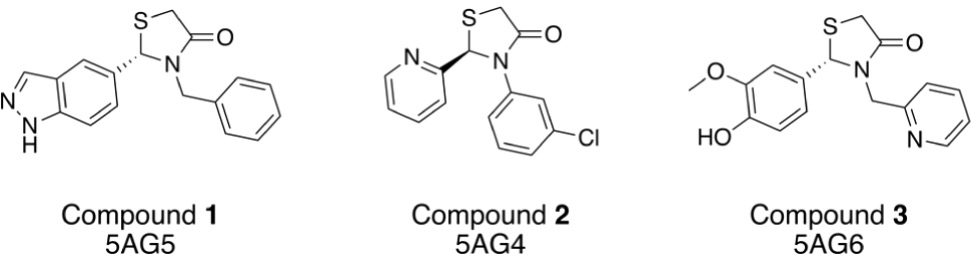
*Lm*NMT thiazolidinone inhibitors and the PDB IDs^53^ corresponding to their X-ray structures with *Lm*NMT.

**Figure 7:**
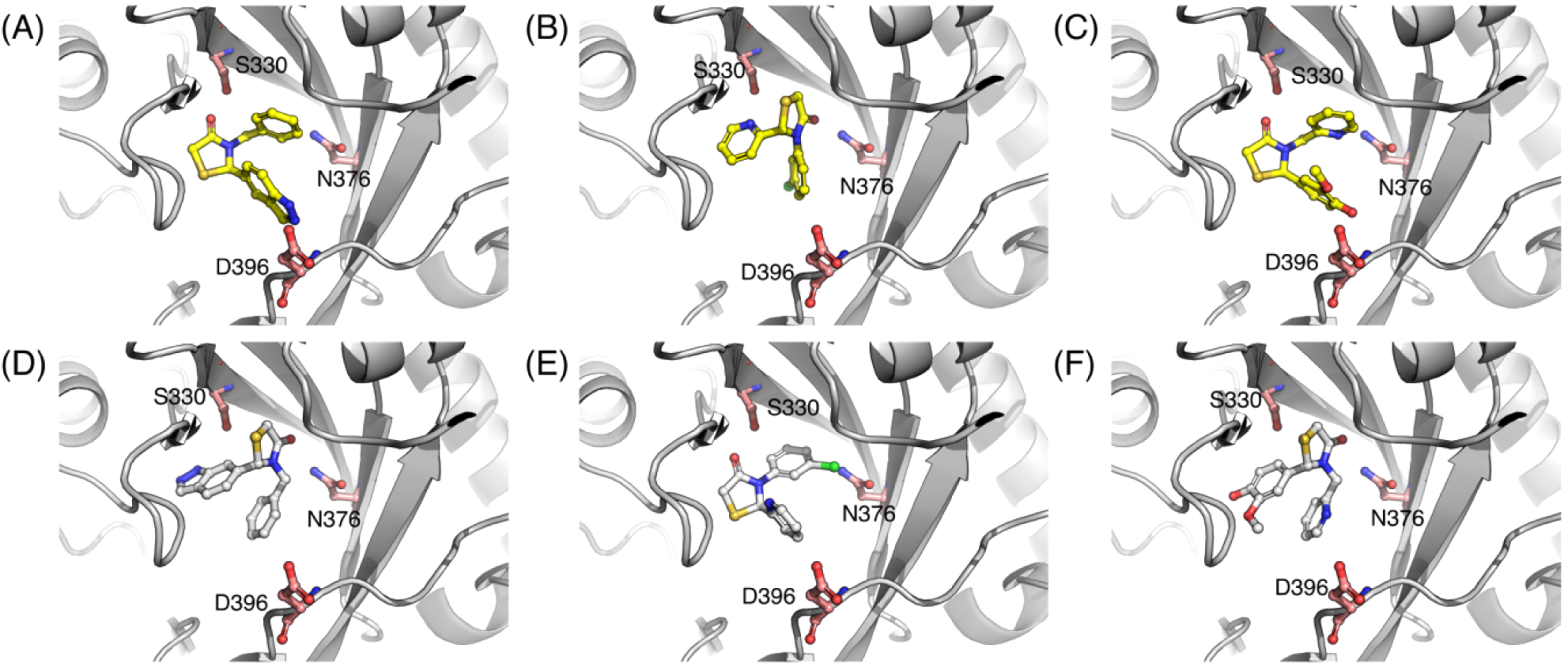
Compounds 1–3 bound to the *Lm*NMT protein from the X-ray structures (top row) and flipped (bottom row) poses: Compound 1 (A, D), compound 2 (B, E) and compound 3 (C, F).

## 4 Simulation Details

### 4.1 System Preparation

#### 4.1.1 Human Neutrophil Elastase (HNE)

The X-ray structure of HNE in complex with compound 1 (Figure 4) was retrieved from the Protein Data Bank (PDB) (PDB ID: 5A8X,^51^ resolution: 2.23 Å). PyMOL^54^ was used to remove the two glycosylations, which are distant from the ligand binding site. The three residues missing in the X-ray structure (Arg146, Asn147, and Arg148) were modeled using the Modeller 9.22 ^55,56^ web service in Chimera.^57^ PropKa3 ^58^ was used for p*K*_a_ predictions, and all amino acids were assigned their standard protonation states at pH 7. Protonation states for histidines were assigned based on visual inspection of their neighboring amino acids (HIS 25, 40, and 57: δ-N; HIS 71 and 210: ε-N). The resulting structure was used for all computations conducted on HNE. The X-ray poses of the compounds were extracted from their respective X-ray structures (after protein structure alignment with the HNE structure from PDB 5A8X) in the PDB (Figure 4) and converted to mol2, pdb, and pdbqt files using Open Babel and PyMOL. The flipped pose of each compound was taken from the molecular docking results (see the Results section).

#### 4.1.2 *N*-Myristoyltransferase (*Lm*NMT)

The X-ray structure of *Lm*NMT with a thiazolidinone (compound 1, Figure 6) was retrieved from the PDB (PDB ID: 5AG5 ^53^, resolution: 2.0 Å). PropKa3 was used for p*K*_a_ predictions; Glu165 was protonated, His299 and His347 were protonated at ε-N, whereas the rest were protonated at δ-N. All the cysteine residues were kept neutral. This final structure was used for all modeling purposes.

The structures of the compounds in their X-ray pose (Figure 7A–C) were taken from their respective PDB structures and converted to the mol2 format (Figure 6). The R enantiomer of compounds 1 and 3 is present in the X-ray structures, whereas for compound 2, the S enantiomer is present. The flipped poses of compounds 1 and 3 (Figure 7D,F) were obtained by taking the X-ray pose of compound 2 (Figure 7B) and replacing the substituents on the thiazolidinone ring with the substituents of compounds 1 and 3, using PyMOL. The resulting structures were minimized using the steepest descent (SD) algorithm in Open Babel (obminimize module) to remove bad contacts. The flipped pose of compound 2 (Figure 7E) was obtained similarly, using the X-ray pose of compound 1 (Figure 7A) and replacing its substituents (no minimization in this case). The stereochemistry of each of the compounds is inverted in the flipped poses (1S, 2R, and 3S) compared to the X-ray poses (1R, 2S, and 3R).

### 4.2 *Lm*NMT Equilibrium Simulations

To define the multiple distance restraints used in the λ-dynamics simulations described in Section 2.2, equilibrium molecular dynamics (MD) simulations were performed, from which the atoms defining the distance restraints were chosen, as described in Section 4.3.

#### 4.2.1 CHARMM36m-CGenFF

The initial ligand coordinates for the X-ray and flipped poses for each compound were taken as described above in the system preparation. The CHARMM36m force field was used for the protein, and the CHARMM general force field (CGenFF) was used for the ligands (Tables 1 and S1). Two separate protein–ligand complexes (one for the X-ray pose and one for the flipped pose), including crystal waters, were formed for each compound using CHARMM.^44,64^ This initial system was minimized using 200 steps of SD in the presence of harmonic position restraints on the protein, followed by 1000 steps of adopted basis Newton–Raphson (ABNR) minimization in the absence of restraints. The system was then solvated using a cubic box of pre-equilibrated TIP3P water molecules, and neutralizing K^+^ ions were added by randomly replacing bulk water molecules. A final minimization was performed employing periodic boundary conditions, particle mesh Ewald (PME)^65,66^ for long-range electrostatics, a nonbonded cutoff of 16 Å along with truncation (VSwitch) of van der Waals interactions between 10 and 12 Å, using 200 steps of SD and 1000 steps of ABNR minimization. The system was then gradually heated from 198 to 298 K with 1 K increments every 100 steps. MD simulations were performed in the isothermal–isobaric ensemble (NPT) at 298 K and 1 atm using a Langevin integrator. The CHARMM/OpenMM^67^ interface was used to accelerate the simulations on graphical processing units (GPUs). The temperature was kept constant using a Langevin heat bath (friction coefficient: 20 ps^–1^ and the pressure was kept constant using the Monte Carlo barostat (move attempts: 25 steps). Heavy atom-hydrogen bond lengths were constrained using SHAKE.^68,69^ A 2 fs integration time step was used for both equilibration (5 ns) and production runs (20 ns).

**Table 1:** Protein and Small-Molecule Force Fields Used along with the Water Model and Ions

System	Protein	Ligand	Water Model	Salt/Ions
HNE	CHARMM36m^59^	CGenFF^60,61^	TIP3P^62,63^	0.15 M KCl
HNE	OPLS-AA^5,6^	OPLS-AA	TIP3P	0.15 M NaCl
*Lm*NMT	CHARMM36m	CGenFF	TIP3P	K^+^
*Lm*NMT	OPLS-AA	OPLS-AA	TIP3P	Na^+^

#### 4.2.2 OPLS-AA

The same starting ligand, protein, and crystal water coordinates were used here as described above. The OPLS-AA force field was used for both the protein and ligands (Tables 1 and S1). The initial system was minimized in the presence (SD: 100 steps; ABNR: 1000 steps) and absence (SD: 100 steps; ABNR: 1000 steps) of harmonic positional restraints on the protein backbone atoms. It was then solvated in a cubic box using pre-equilibrated water molecules (TIP3P) and neutralized by replacing bulk water molecules with Na^+^ neutralizing ions. The final system was then minimized in the presence of periodic boundary conditions, with PME, a nonbonded cutoff of 16 Å, along with truncation of van der Waals (VFSwitch) between 10 and 12 Å, using 200 steps of SD and 1000 steps of ABNR. The same procedure as described above was followed for heating, equilibration (5 ns), and production (10 ns) MD simulations. The production simulations were kept shorter than those with CHARMM36m-CGenFF since 10 ns appeared sufficient to generate the statistics for multiple distance restraints.

### 4.3 Multiple Distance Restraints for *Lm*NMT

The equilibrium MD trajectory was used to pick the multiple distance restraints between the protein and the X-ray poses. All the protein heavy atoms that were between 10 and 15 Å away from every ligand heavy atom were selected to calculate the average distances over the whole trajectory. For each ligand heavy atom-protein heavy atom(s), the pair with the lowest standard deviation was kept. Then, only the unique ligand-protein atom pairs were retained, ensuring that each ligand or protein heavy atom was involved in only one restraint. The restraints were added using the NOE module in CHARMM. The *R*_min_ and *R*_max_ values for the NOE restraints were set based on the average distance *d*_average_ (*R*_min_ = *d*_average_ – 4 Å; *R*_max_ = *d*_average_ + 4 Å) obtained from the MD trajectory for a particular protein–ligand atom pair. For the flipped poses, however, our preliminary analysis of the MD trajectory showed large deviations from the starting structure (Figures S2 and S3). So, to conserve the starting flipped mode, we instead used the final minimized structure to pick the multiple distance restraints using the same procedure described above for the X-ray pose. The protein and ligand atoms selected for restraints are shown in Figures S4 and S5 (and listed in Tables S4 and S5).

The final minimized structure of the *Lm*NMT-flipped pose complex was superimposed on the final frame of the MD simulation of *Lm*NMT-X-ray complex using MDAnalysis v2.0.0.^70,71^ The coordinates of the flipped pose were extracted and used, along with the X-ray structure of the *Lm*NMT complex, for λ-dynamics and OSP simulations.

### 4.4 λ-Dynamics Calculations

Individual dual-topology systems were defined for all of the HNE ligands, consisting of each ligand in its X-ray and flipped poses. The initial ligand coordinates were taken from the X-ray poses by aligning the protein in all PDB files with the protein in 5A8X using PyMOL. The coordinates for the flipped poses were obtained from the docked structures. Coordinate and topology files for the HNE-ligand complexes were generated using CHARMM. Two different force field combinations were used for parameterizing the protein and ligands (Tables 1 and S1). Each protein–ligand complex system was solvated with TIP3P water molecules. A cubic box was defined such that it extended 10 Å from the longest axis of the protein–ligand complex. To neutralize the system, K^+^ (or Na^+^) and Cl^–^ ions were added, corresponding to a 0.15 M KCl (or NaCl) concentration. Similarly, dual-topology systems were defined for *Lm*NMT and its ligands, using the coordinates from the equilibrium MD simulations (Sections 4.2 and 4.3) and two different force fields (Table 1). For both the HNE and *Lm*NMT cases, a one-site MSλD system containing two substituents (substituent 1 – X-ray pose and substituent 2 – flipped pose) was defined using the BLOCK module in CHARMM. All angles and dihedrals between alchemical groups were deleted. The default functional form of λ^72^ (with an FNEX value of 5.5) was used. Bonds, angles, and impropers were excluded from scaling with λ. ALF biases were assigned to both substituents within the CHARMM BLOCK. Nonbonded interactions between the two substituents were excluded. CHARMM NOE-based tethering was used in the case of HNE between the ligand poses to avoid wandering off using a force constant of 100–150 kcal/mol/Å^2^. The ligand atoms were chosen by visual inspection in PyMOL. A total of 4–8 (2 or 4 for each pose) atoms were picked from each ligand pose. Two heavy atoms were picked from the dihydropyrimidinone ring, one atom (meta-carbon) from the trifluoromethyl phenyl ring, and the fourth atom (para-carbon) from the cyanophenyl ring for each of the two poses. In the case of *Lm*NMT, multiple protein–ligand distance restraints were used and were defined as described in Section 4.3. The NOE module in CHARMM was used for multiple distance restraints with a force constant of 40 or 50 kcal/mol/Å^2^.

All the λ-dynamics calculations were performed using the CHARMM package (47a2 or 49a1) on graphical processing units (GPUs) using BLaDE.^73^ Simulations of both the HNE and *Lm*NMT systems with the OPLS-AA force field were specifically performed using the 49a1 version of CHARMM/BLaDE, since this interface now supports the use of force field nonunit values for e14fac (OPLS-AA: 0.5) and geometric van der Waals combination rules. All the systems were minimized using 200–400 steps of SD in the presence of periodic boundary conditions, particle mesh Ewald (PME) for long-range electrostatics, a nonbonded cutoff of 12 Å, along with truncation (VFSwitch) of van der Waals interactions between 9 and 10 Å. MD simulations were performed in the isothermal–isobaric ensemble (NPT) at 298.15 K and 1 atm. BLaDE used a Monte Carlo barostat for pressure coupling (move attempts: 100 steps) and a Langevin thermostat for temperature coupling (friction coefficient: 0.1 ps^–1^. A 2 fs integration time step was used to integrate the equations of motion, and hydrogen bond lengths were constrained using SHAKE. λ values were saved every 20 fs, and soft-core potentials were used to avoid endpoint singularities.

The λ-dynamics simulations consist of several steps, where initial estimates of the ALF biases (coefficients ϕ,ψ,ω,χ ; cf. Supporting Information) are first obtained by running several 100 ps long simulations, followed by 10–20 simulations of 1 ns duration each to optimize the ALF biases. Once the ALF biases were optimized and the λ landscape had flattened, we ran 5 equilibration replicas of 5 ns each, followed by production simulations in 5 replicates of 30 ns each. The free energy estimates and the associated uncertainties are obtained from the 5 independent production runs using the weighted histogram analysis method (WHAM)^74^ and bootstrapping, respectively, where the first 5 ns are usually discarded.

### 4.5 Multisite λ-Dynamics Calculations

A multiple topology model (MTM) was defined for all five HNE ligands (compounds 1–5) using msld_py_prep^75^ resulting in a two-site system. The resulting hybrid ligand contained renormalized charges (CRN) to keep the sum of core charges and the charge on substituents at the two sites neutral. One copy of the hybrid ligand was solvated in a water box using MMTSB toolset^76^ and the other copy was merged with the protein structure, followed by solvation and neutralization using the MMTSB toolset. All the other MSλD parameters, water model, salt concentration, and simulation parameters were kept the same as defined in Section 4.4 (unless stated otherwise) for HNE. The simulations were run using the CHARMM package v49a1 on GPUs, using only the OPLS-AA force field for both the ligands and the protein. Two independent repeats were carried out: one by using a scaling factor of 0.5 for the nonbonded 1–4 electrostatic interactions (e14fac, default for OPLS-AA) and the other by using a factor of 1.0, meaning no scaling was applied. ALF flattening was run for both the water and complex sides, but the complex side flattening runs were initiated with the flattened biases obtained from water-side simulations. This strategy ensures rapid convergence of ALF biases for the complex side and reduces overall computational expense (Table S15). After the ALF flattening runs, equilibration simulations were run for 25 ns (5 replicas ∗ 5 ns each) for both the water and the complex sides. The water side production runs were carried out for 125 ns (5 replicas ∗ 25 ns each), and the complex side production simulations were run for 200 ns (5 replicas ∗ 40 ns each). The free energies obtained from MSλD calculations were with renormalized charges (CRN), and to obtain the free energy differences with original force field (FF) charges, bookending charge correction simulations using single-step perturbation were carried out. The charge-corrected relative binding free energies were then converted to absolute binding free energies. A detailed explanation of how to obtain bookending charge corrections and charge-corrected absolute binding free energies can be found in our previous work.^52^

### 4.6 Contribution of Multiple Distance Restraints in *Lm*NMT Using OSP

The starting structures for the reference state in Figure 2 were the same as those used for λ-dynamics simulations. The BLOCK module in CHARMM was again used to define the two substituents, and the MSλD module was used with fixed λ values (FFIX). Both poses were assigned their individual λ’s ( $\lambda_{xray}=0.5\;and\;\lambda_{flip}=0.5$ ). The MD simulations of the reference state were run using CHARMM with the same conditions and parameters as those used for the λ-dynamics simulations. Three independent simulations (with different random seeds for velocity assignment) of 5 ns each were run. The trajectories generated from the reference state were postprocessed with CHARMM using the λ’s and force constants corresponding to each of the end states in Figure 2 to obtain the energies corresponding to the end states for use with Zwanzig’s equation. An overview of the overall workflow for *Lm*NMT is given in Figure 8.

**Figure 8:**
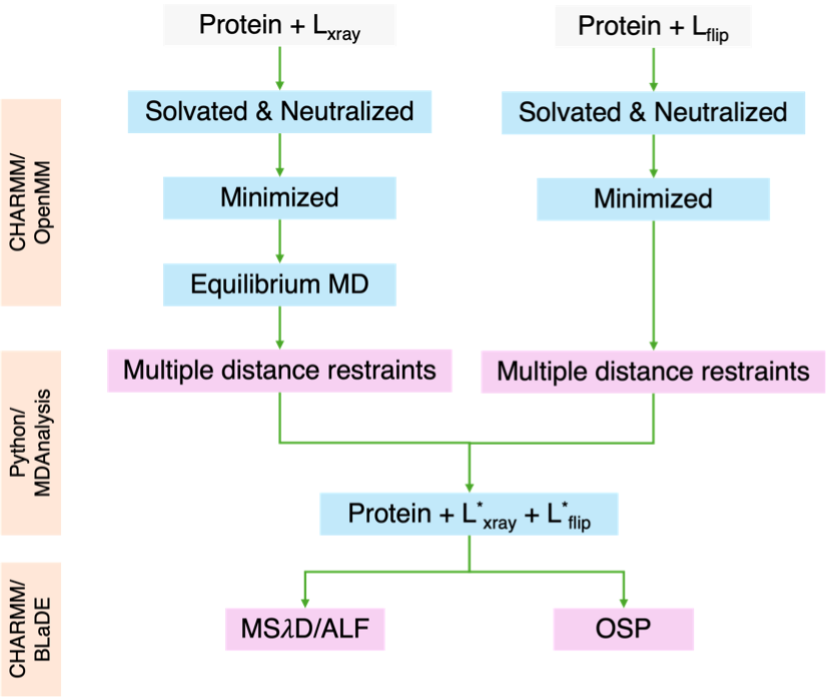
Workflow combining relative binding free energy calculations using MSλD and endpoint corrections for removing/adding restraints using the one-step perturbation (OSP) approach for accurately ranking the binding of flipped poses. Equilibrium MD consists of three steps; gradual heating, equilibration run, and production run.

### 4.7 Analysis

All of the trajectories were processed with CHARMM. VMD^77^ was used to visualize the simulation trajectories. PyMOL was used to generate figures and edit small molecule structures. MDAnalysis 2.0.0 was used to compute root-mean-square distances from simulation trajectories and to align/superimpose structures.

## 5 Results and Discussion

### 5.1 Molecular Docking

#### 5.1.1 HNE

Docking with AutoDock Vina resulted in the X-ray pose being predicted as the top pose only for compounds 1 and 3 (Table S2). The top-ranked pose for compound 2 was a flipped pose (Figure 5B) where the trifluoromethyl phenyl group is bound in the S2 pocket, and the cyanophenyl group is bound in the S1 pocket. For compounds 1 and 3, the second top-ranked pose was the same flipped pose, and the predicted binding affinity relative to the X-ray pose was only 1 and 1.6 kcal/mol, respectively (Table S2). The X-ray poses of compounds 4 and 5 could not be reproduced with AutoDock Vina. For those compounds, the obtained poses resulted in the compounds being docked in either the S1 or S2 pocket, but not in both pockets as in the X-ray structure. Using another scoring function did not improve the outcome for compounds 4 and 5; Smina predicted the X-ray pose for compound 4 as the third-ranked pose and the flipped pose for compound 5 as the fourth-ranked pose. In the top-ranked poses, the compounds were again docked in either the S1 or S2 pocket, but not both.

While the ligand binding mode from the X-ray structures is unambiguous, the docking results for compounds 1–5 in HNE illustrate the difficulty for scoring functions to distinguish and correctly rank X-ray and other poses in a congeneric series. The heterogeneity of the binding poses predicted for the 5 congeners makes this system a relevant test case for testing the ability of our RBFE scheme to rank, for each compound, the X-ray pose and the flipped pose (obtained from docking).

#### 5.1.2 *Lm*NMT

The X-ray structures of *Lm*NMT for compounds 1–3 show core-flipping in the congeneric series (Figure 7). Interestingly, molecular docking did not predict the X-ray pose as the top pose for any of the compounds, and the X-ray pose was among the top five predicted poses only for compound 1 (ranked second) and for compound 3 (third ranked top pose) (Table S3). For those two compounds, the flipped pose, i.e., the X-ray pose of compound 2, was not within the five best poses. For compound 2, neither the X-ray pose nor the flipped pose (i.e., the X-ray pose for compounds 1 and 3) was within the five best poses. The discrepancy between docking and X-ray is likely due to the shallow and solvent-exposed binding pocket, which is difficult for the search algorithm to navigate. The soft degrees of freedom, i.e., the rotatable torsions of the ligands, are an additional challenge for rank ordering by the scoring function.

### 5.2 RBFE for Ligand Poses in HNE

The relative binding free energy (RBFE) of the flipped pose compared to the X-ray pose for compounds 1–5 is reported in Table 2. For each of the five compounds, the flipped pose is predicted to be highly unfavorable (>9.5 kcal/mol) compared to the corresponding X-ray pose, in agreement with the structural data available. The free energies obtained from MSλD were converged in all cases. Restraining the ligand poses to one another maintained the binding mode for both poses, and no wandering-off was observed. λ-dynamics calculations for these sets of compounds consisted of around 25 heavy atom perturbations for each pose. The ALF fixed biases were flattened in all cases, and the overall free energy landscape was also converged (cf. Figure S6). The total number of transitions per nanosecond between the ligand poses and the fraction of physical ligand is low because of the large size of the perturbation (Table S6). This is also reflected in the high barriers observed in the λ landscapes in Figure S6. Overall, this system was handled well by MSλD/ALF, and no issues related to sampling, ligand wandering off, or convergence were observed. The X-ray poses of each of the compounds were correctly predicted as energetically more favorable than their flipped poses.

**Table 2:** MSλD Relative Binding Free Energy Differences between the Flipped and X-Ray Poses of Compounds 1–5 to HNE; Values Obtained Using eq 4

	$\Delta \Delta G_{xray\to flip}$ (kcal/mol)		
Compound	CHARMM-CGenFF	OPLS-AA	Match with X-ray
1	10.4 ± 0.6	3.3 ± 0.1	yes
2	12.5 ± 0.1	13.2 ± 0.2	yes
3	11.1 ± 0.1	8.9 ± 0.1	yes
4	14.2 ± 0.2	12.8 ± 0.2	yes
5	9.5 ± 0.1	7.7 ± 0.3	yes

We repeated these calculations with the OPLS-AA force field to check the sensitivity to different FFs. The corresponding relative predicted binding free energies are also reported in Table 2. The free energies were converged in all cases, and the X-ray pose is favored over the flipped pose for all compounds. The λ landscape for all the five compounds was converged (cf. Figure S7) and the fixed biases were flattened (cf. Figure S7). The high barriers in the λ landscape were again observed, in agreement with the CHARMM36m-CGenFF force field combination, resulting in low transitions between the two poses and a low fraction of physical ligand (cf. Table S6).

### 5.3 MSλD Predictions for the Congeneric Series of HNE Using the Favorable Poses from λ-Dynamics Calculations

Following the results from the previous section, we took the more favorable pose (in this case, the X-ray pose) for each of the five HNE ligands and calculated their relative and absolute binding free energies using the OPLS-AA force field and compared them to the experimental activity data from Nussbaum et al.^50,51^ (see Tables S8 and S9). Using the absolute binding free energies calculated from the X-ray poses ( $\Delta G_{bind}^{xray}$ ) and the relative binding free energy differences between the flipped and X-ray poses ( $\Delta \Delta G_{xray\to flip}$ , Table 2), we calculated the absolute binding free energies using the flipped poses ( $\Delta G_{bind}^{flip}$ , Table S10). The $\Delta G_{bind}^{flip}$ values correlate poorly with the experimental affinities, as shown by a root-mean-square error (RMSE) of 9.5 kcal/mol (Pearson’s correlation coefficient *R* = −0.5). The corresponding RMSE using the X-ray poses is only 1.5 kcal/mol (*R* = 0.77), and the computed free energies correlate well with the experimental data, reaffirming the choice of the favorable pose predicted from the λ-dynamics calculations. It is to be noted that the difference in chemical structure between compounds 1–5 is quite large, explaining the somewhat large RMSE obtained, but we have shown earlier that it yields higher accuracy in congeneric series with smaller differences between compounds.^52^ Also, the RMSE is improved to 1.0 kcal/mol by using an e14fac value of 1.0 instead of 0.5.

### 5.4 RBFE for Ligand Poses in *Lm*NMT

The relative binding free energies of the flipped poses compared to their X-ray poses ( $\Delta \Delta G_{xray\to flip}$ ) using the CHARMM36m-CGenFF force field combination, are reported in Table 3. Compounds 2 and 3 are correctly predicted to bind more favorably in their X-ray poses than in the flipped poses, but this is not the case for compound 1, which is predicted to bind more favorably in the flipped pose. However, the relative binding free energy is low (−2.5 kcal/mol) and the uncertainty is high (1.2 kcal/mol), making the results inconclusive for distinguishing the X-ray pose from the flipped pose. The free energies from MSλD were converged, but the ALF fixed bias did not flatten, even though the overall λ landscape was converged in all cases. The ALF free energy λ landscape and the evolution of different bias coefficients are shown in Figure S8. A low number of transitions per nanosecond between the substituents (Table S7) was observed, which translates to a high fraction of physical ligand.

**Table 3:** MSλD Relative Binding Free Energy Differences (in kcal/mol) between the Flipped and X-ray Poses of Compounds 1–3 to *Lm*NMT ( $\Delta \Delta G_{xray\to flip}$ , eq 10 and Figure 3)­^a^ ^b^

	CHARMM36m-CGenFF				
Compound	$$\Delta G_{xray^{**}\to flip^{**}}^{MS\lambda D}$$	$$\Delta G_{xray\to xray^{**}}^{OSP}$$	$$\Delta G_{flip^{**}\to flip}^{OSP}$$	$$\Delta \Delta G_{xray\to flip}$$	Match with X-ray
1	–2.1 ± 0.8	0.8 ± 0.4	–1.2 ± 0.8	–2.5 ± 1.2	no
2	11.2 ± 0.3	1.1 ± 0.4	–1.3 ± 0.2	10.9 ± 0.4	yes
3	1.7 ± 0.1	1.2 ± 0.4	–1.0 ± 0.1	1.9 ± 0.4	yes

a The contributions of multiple distance restraints are also provided.

b All values were obtained using the CHARMM36m force field for the protein and CGenFF for the ligands.

We next repeated the same calculations using the OPLS-AA force field for both the protein and ligand. The relative binding free energies obtained with OPLS-AA are reported in Table 4. For all three compounds, the X-ray pose is predicted to be highly favorable compared to the flipped pose. The free energy differences from MSλD are well-converged with low uncertainties (except for compound 2). The overall λ landscape between the substituents from ALF was again converged (Figure S9) and the substituents were fairly sampled in all cases so that we could extract the free energies. Using the OPLS-AA force field thus improves the predictive power of our protocol for this particular system.

**Table 4:** MSλD Relative Binding Free Energy Differences (in kcal/mol) between the Flipped and X-ray Poses of Compounds 1–3 to *Lm*NMT ( $\Delta \Delta G_{xray\to flip}$ , eq 10, Figure 3)­^a^

	OPLS-AA				
Compound	$$\Delta G_{xray^{**}\to flip^{**}}^{MS\lambda D}$$	$$\Delta G_{xray\to xray^{**}}^{OSP}$$	$$\Delta G_{flip^{**}\to flip}^{OSP}$$	$$\Delta \Delta G_{xray\to flip}$$	Match with X-ray
1	10.2 ± 0.1	0.8 ± 0.6	–1.1 ± 0.2	9.9 ± 0.6	yes
2	6.5 ± 0.9	1.3 ± 0.3	–1.2 ± 0.4	6.6 ± 1.0	yes
3	7.4 ± 0.1	1.5 ± 0.7	–1.5 ± 0.2	7.4 ± 0.7	yes

a Values are obtained using the OPLS-AA force field for both the protein and the ligands.

Overall, the RBFE calculations for this test system proved more challenging than the HNE system because of the fact that the ligand binding site of *Lm*NMT is shallow and large compared to the size of the compounds bound. Using NOE-based tethering of the ligand poses to one another, as used in the case of HNE, did not yield sufficient sampling, and in some cases, the ligands wandered off in the simulation box. We therefore used multiple protein–ligand distance restraints to avoid sampling issues and the ligand from wandering off the compounds. Figures 9–11 show snapshots for all the compounds during the λ-dynamics simulations with both CHARMM-CGenFF and OPLS-AA force fields. The snapshots were generated from λ-dynamics simulation frames where a particular pose was dominant (λ > 0.99). The thiazolidinone ring of the core in simulations of both the X-ray and flipped poses matched their starting pose (Figure 7) and was oriented similarly across the two force fields.

**Figure 9:**
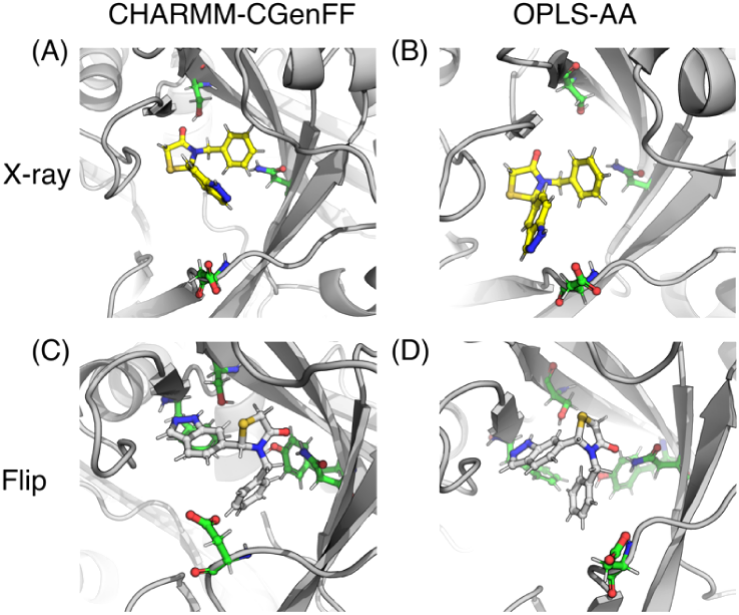
Snapshots from λ-dynamics simulations for compound 1. (A) X-ray pose and (C) flipped pose using CHARMM-CGenFF force field combination. (B) X-ray pose and (D) flipped pose using the OPLS-AA force field.

**Figure 10:**
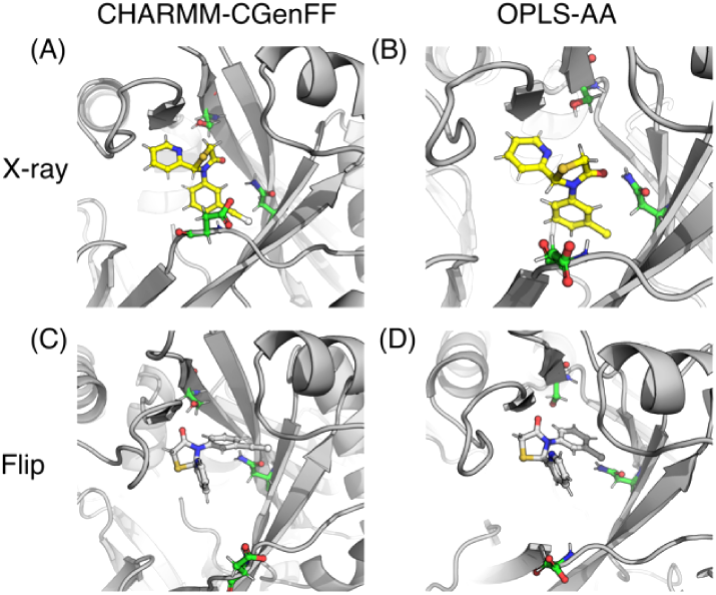
Snapshots from λ-dynamics simulations for compound 2. (A) X-ray pose and (C) flipped pose using CHARMM-CGenFF force field combination. (B) X-ray pose and (D) flipped pose using the OPLS-AA force field.

**Figure 11:**
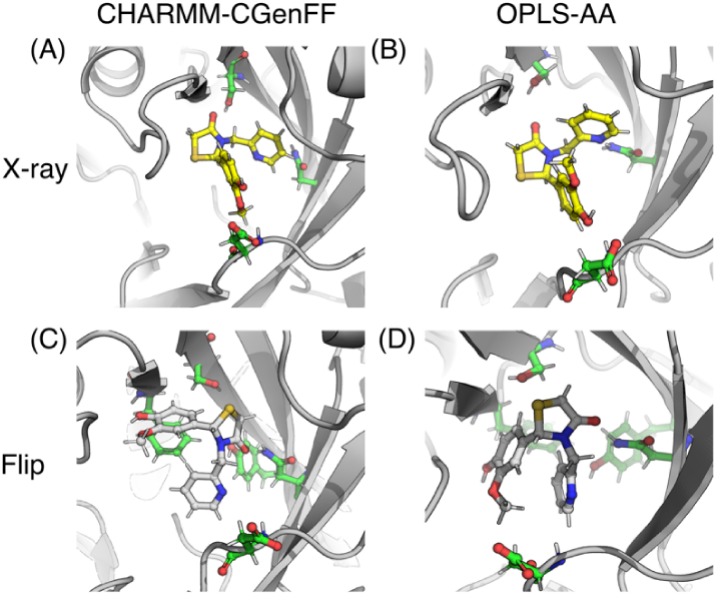
Snapshots from λ-dynamics simulations for compound 3. (A) X-ray pose and (C) flipped pose using CHARMM-CGenFF force field combination. (B) X-ray pose and (D) flipped pose using the OPLS-AA force field.

The $\Delta \Delta G_{xray\to flip}$ values obtained for compound 1 with CHARMM-CGenFF and OPLS-AA are strikingly different, with −2.5 ± 1.2 kcal/mol and 9.9 ± 0.6 kcal/mol, respectively, and the difference is largely explained by the values of $\Delta G_{xray^{**}\to flip^{**}}^{MS\lambda D}$ (−2.1 ± 0.8 vs 10.2 ± 0.1 kcal/mol). Looking at the point charges of the compounds and the protein residues in the first interaction shell, we could not identify any important difference between the force fields. Likewise, the inventory of protein–compound polar interactions (Table S11) and hydrogen bonds (Table S12) in dominant frames of the λ-dynamics simulations does not provide a simple explanation for the difference in $\Delta G_{xray^{**}\to flip^{**}}^{MS\lambda D}$ between the two force fields.

### 5.5 Comparing the OSP and Windowing Approaches to Remove Multiple Distance Restraints in *Lm*NMT

We also used the windowing approach to calculate the free energy cost associated with the multiple distance restraints between the protein and compound 2 poses, using the CHARMM-CGenFF force field combination (cf. Supporting Information). The results are reported in Table S13. Using the FEP/MBAR approach was not only computationally more expensive than the OSP approach discussed above but also led to ligand wandering off and convergence issues. The noninteracting pose wandered off upon turning the restraints fully off (Figures S11 and S12). This led to poor phase space overlap between the penultimate window and the last window (Figure S13) and caused convergence issues for MBAR (Figure S14). Comparatively, in the OSP approach, the fluctuations in the energies on going from the reference state to both endpoints in the backward and forward direction were not large, and the free energies for individual replicas converged well (Figures S15 and S16). The different replicas of the OSP simulations also sampled different parts of the configurational space. The root-mean-square fluctuations (RMSF) of the ligand poses (both with λ = 0.5) from three independent replicas are shown in Figure S17. However, the statistical uncertainties for $\Delta G_{xray\to xray^{**}}^{OSP}$ and $\Delta G_{flip^{**}\to flip}^{OSP}$ (Tables 3 and 4) with OSP are higher than with FEP/MBAR, and they are on occasion also higher than the uncertainties in the λ-dynamics calculations. This can be attributed to the larger size of the protein binding pocket compared to the size of the compounds combined with the definition of the OSP reference state.

### 5.6 Computational Resources

The λ landscape was flattened in a short simulation time for both HNE and *Lm*NMT (11–30 ns; cf. Tables S14 and S16). A typical run for HNE simulations on the hardware we used (Intel Core i7–9700F CPU and NVIDIA GeForce RTX 3080 Ti) could produce 250 ns/day. The same hardware for *Lm*NMT simulations could produce around 111 ns/day. Newer hardware (AMD Ryzen 9 7900X and NVIDIA GeForce RTX 4090) could accelerate the *Lm*NMT simulations further, producing around 180 ns/day. We hypothesize that the inclusion of more than 2 poses in a single λ-dynamics simulations would consume a similar number of resources, thus improving the efficiency of these calculations with an increasing number of alternate poses.

## 6 Conclusion

Accurate prediction of poses of ligands in a congeneric series is decisive for the establishment of the SAR in hit-to-lead and lead optimization phases. Yet, it is challenging to accurately rank flipped ligand poses using docking. Here, we present a simple and modular workflow to accurately rank two alternative poses of small molecule inhibitors in a congeneric series. The workflow is based on RBFE calculations using λ-dynamics in combination with restraints and a dual-topology model. It was tested on two different pharmaceutically relevant drug targets: HNE and *Lm*NMT. Experimental structural data are available for *Lm*NMT bound to three ligands in a congeneric series and for five ligands bound to HNE. Docking led to a poor prediction of the binding poses, but using our RBFE protocol, we were able to correctly rank the flipped pose compared to the X-ray pose in both cases. Because of the shallow and large ligand binding pocket of *Lm*NMT the calculations were more challenging than those with HNE, and their accuracy was sensitive to the force field.

This workflow can be particularly useful for prospective drug design, which lacks experimental structural data and consensus from docking-predicted poses. In such cases, the alternate poses from docking can be reranked with λ-dynamics. This model/pose can be used for performing RBFE calculations and combined with in vitro activity data to further strengthen the choice of the starting orientation of the core structure for lead optimization. As a retrospective validation, we used the most favorable poses obtained from our λ-dynamics calculations to perform RBFE calculations for the HNE ligands and obtained good agreement with the experimental data, while the agreement was poor when using the least favorable poses. In general, ranking more than two poses (e.g., those predicted by docking programs) in a single simulation would follow a procedure similar to the one proposed here for two poses. However, further investigation of the achieved convergence and sampling with the current version of ALF is required. The proposed methodology can easily be extended and applied to alternative rotamers of amino acids in ligand binding sites or for ranking different scaffolds that do not possess a common core, such as in the case of scaffold hopping or fragment-based drug design.

## Supplementary Material



## Data Availability

All data and software used in this study are freely available. Data identifiers and sources are given in the text. λ-dynamics and OSP simulation files are available at https://github.com/reuter-group/core_flipping. The repository contains the MSλD and OSP prep directories, ALF production variables, CHARMM input scripts used for calculating the relative binding free energies, and starting protein and ligand structures for MD simulations in the case of *Lm*NMT and the corresponding CHARMM input scripts.

## Notes

Details for small molecule parameters, molecular docking, windowing approach for removing restraints; results of docking, ligand RMSD from equilibrium MD simulations, ALF convergence plots, MSλD binding free energy predictions for HNE, interaction analysis, windowing approach, OSP simulation convergence analysis, ligand RMSF from the OSP methodology, and simulation times (PDF)
