# Supplementary material for: Core Flipping in Lead Optimization: Rank Ordering Using λ‑Dynamics
Source: J Chem Inf Model. 2025 Jun 16;65(13):6835–46. doi: 10.1021/acs.jcim.5c00320 (PMC12264938; doi:10.1021/acs.jcim.5c00320)
Supplement: Supplementary file 1 [file ci5c00320_si_001.pdf]

Supporting Information

Core Flipping in Lead Optimization: Rank Ordering using λ-dynamics

Parveen Gartan[1,2], Charles L. Brooks III[3,4*], Nathalie Reuter[1,2*]

[1]Department of Chemistry, University of Bergen, Bergen, 5020, Norway

[2]Computational Biology Unit, University of Bergen, Bergen, 5020, Norway

[3]Department of Chemistry, University of Michigan, Ann Arbor, Michigan, 48109, USA

[4]Biophysics Program, University of Michigan, Ann Arbor, Michigan, 48109, USA

* Corresponding authors: brookscl@umich.edu, nathalie.reuter@uib.no

1. Methods

1.1 Small Molecule Parameters. Parameters for selected compounds were generated using the programs mentioned in **Table S1**.

Table S1. Programs used to generate small molecule parameters.

System	Ligand	Program	Force Field
HNE	CGenFF	CGenFF 2.5.1[1,2] (Silcsbio 2022.1)	CGenFF 4.1
HNE	OPLS-AA	LigParGen[3] with BOSS 5.0	1.14*CM1A-LBCC/OPLS-AA
*Lm*NMT	CGenFF	CGenFF 2.5.1 (Silcsbio 2022.1) or 3.0 (Silcsbio 2024.1)	CGenFF 4.1
*Lm*NMT	OPLS-AA	LigParGen[3] with BOSS 5.0	1.14*CM1A-LBCC/OPLS-AA

1.2 Molecular Docking. Autodock Vina[4] was used to dock the selected compounds for both HNE and *Lm*NMT systems. Docking with Smina[5] was also performed for HNE compounds. The protein pdbqt files were prepared using Autodock Tools (HNE structure from PDB ID: 5A8X; *Lm*NMT structure from PDB ID: 5AG5; see section 4.1 main text). Polar hydrogens were added to the protein. A grid box (dimensions 20*20*20, spacing 1 Å) was defined for each of the two proteins such that it encompasses the binding site. It was centered on the HIS57 δ-N atom for HNE and on TYR345 OH atom (CHARMM36 FF nomenclature) for *Lm*NMT. A default exhaustiveness of 8 was used with Vina. Smina was utilized using the vinardo scoring function, an exhaustiveness of 20 and an energy range of 10 kcal/mol.

1.3 FEP/MBAR to Remove Multiple Distance Restraints to *Lm*NMT. The same starting structure was used here as in λ-dynamics and OSP simulations. The BLOCK and MSλD modules were used with fixed λ values (FFIX). All the molecular dynamic simulations parameters were similar as in the λ-dynamics and OSP simulations. The end-point states are defined as shown in **Figure S1**. For removing the multiple distance restraints from the X-ray pose, we take the X-ray pose with λ=1 and the flip pose with λ=0. For removing the multiple distance restraints from the flipped pose, the X-ray pose was kept at λ=0 and flip pose at λ=1. The NOE force constant was gradually decreased from 40 kcal/mol/Å^2 to 0

using 16 windows. All the windows were run for 5 ns each and the simulation trajectories were then postprocessed with CHARMM. FastMBAR[6] was used to calculate the free energy differences on going from the first window to the penultimate window. Zwanzig's perturbation formula was used to calculate the unidirectional free energy of going from the penultimate window to the final window. The free energy of removing (or adding) the restraints were combined with the free energy from MSλD to get the final free binding free energy of the flipped pose compared to the x-ray pose using equation **S1**.

$$\Delta G_{xray \rightarrow flip} = \Delta G^{FEP/MBAR}_{xray \rightarrow xray^{**}} + \Delta G^{MS\lambda D}_{xray^{**} \rightarrow flip^{**}} + \Delta G^{FEP/MBAR}_{flip^{**} \rightarrow flip} \tag{S1}$$

$$\begin{matrix} Xray\ (\lambda = 1) \\ Flip\ (\lambda = 0) \end{matrix} \xrightarrow{\Delta G^{FEP/MBAR}_{xray \rightarrow xray^{**}}} \begin{matrix} Xray^{**}\ (\lambda = 1) \\ Flip^{**}\ (\lambda = 0) \end{matrix} \xrightarrow{\Delta G^{MS\lambda D}_{xray^{**} \rightarrow flip^{**}}} \begin{matrix} Xray^{**}\ (\lambda = 0) \\ Flip^{**}\ (\lambda = 1) \end{matrix} \xrightarrow{\Delta G^{FEP/MBAR}_{flip^{**} \rightarrow flip}} \begin{matrix} Xray\ (\lambda = 0) \\ Flip\ (\lambda = 1) \end{matrix}$$

Figure S1. Thermodynamic correction for calculating the free energy change of flipped pose compared to the X-ray pose from MSλD with multiple distance restraints. FEP/MBAR is used to calculate the contribution for adding/removing the restraints. ** - represents the ligand multiple distance restraints to protein with the full value of force constant (in kcal/mol/Å^2).

1.4 Adaptive Landscape Flattening (ALF). ALF is a set of biases that are used in λ-dynamics simulations to flatten the λ landscape between different substituents which differ more than 2-3 kcal/mol from each other. ALF consists of four bias terms which include fixed, quadratic, endpoint, and skew bias.

$$\Delta G = \phi_1(1-\lambda) + \phi_2\lambda + \psi_{12}\lambda(1-\lambda) + \frac{\omega_{12}\lambda(1-\lambda)}{\lambda+\alpha} + \frac{\omega_{21}\lambda(1-\lambda)}{(1-\lambda)+\alpha} + \chi_{12}(1-\lambda)\left(1-\exp\left(\frac{-\lambda}{\sigma}\right)\right) + \chi_{21}\lambda\left(1-\exp\left(\frac{-(1-\lambda)}{\sigma}\right)\right) \tag{S2}$$

where,

$$\sigma = 0.18$$

$$\alpha = 0.017$$

$$\phi\ (or\ lam) = fixed\ bias$$

$$\psi\ (or\ c) = quadratic\ bias$$

$$\omega\ (or\ s) = end\ point\ bias$$

$$\chi \ (or\ x) = skew\ bias$$

1.5 Volume Correction. The free energy obtained from **equation 10** (see main text; also see **equations 8** and **9**) does not include the cost of restraining the non-interacting ligand to sample only the binding site of the protein. In an ideal case (or case of infinite sampling), the non-interacting pose/ligand should sample the entire volume of the simulation box. This correction to the final free energy for the flipped and X-ray pose is given below.[7,8]

For the non-interacting flipped pose we have (left part; **Figure 3** in main text),

$$flip(\lambda = 0) \rightarrow flip^{**}(\lambda = 0)$$

The volume correction can be written as:

$$\Delta G_{vol} = -k_B T ln\left(\frac{V_{flip^{**}(\lambda=0)}}{V_{flip(\lambda=0)}}\right) \qquad (S3)$$

$$\Delta G_{vol} = -k_B T ln\left(\frac{V_{flip^{**}(\lambda=0)}}{V_{box}}\right) \qquad (S4)$$

Similarly, for the non-interacting X-ray pose we have (right part; **Figure 3** in main text),

$$xray^{**}(\lambda = 0) \rightarrow xray(\lambda = 0)$$

And the volume correction can be written as:

$$\Delta G_{vol} = -k_B T ln\left(\frac{V_{xray(\lambda=0)}}{V_{xray^{**}(\lambda=0)}}\right) \qquad (S5)$$

$$\Delta G_{vol} = -k_B T ln\left(\frac{V_{box}}{V_{xray^{**}(\lambda=0)}}\right) \qquad (S6)$$

The total contribution to the free energy for both of these poses can be written as:

$$\Delta G_{vol}^{corr} = -k_B T ln\left(\frac{V_{flip^{**}(\lambda=0)}}{V_{box}} * \frac{V_{box}}{V_{xray^{**}(\lambda=0)}}\right) \qquad (S7)$$

$$\Delta G_{vol}^{corr} = -k_B T ln\left(\frac{V_{flip^{**}(\lambda=0)}}{V_{xray^{**}(\lambda=0)}}\right) \qquad (S8)$$

In our case, the two poses of a given ligand (X-ray and flipped) sample similar volume space in the binding site so, the volume ratio in above equation should become negligible or close to 1.

2. Results

2.1 Molecular docking

Table S2. Results from docking of compounds 1-5 with HNE.

Compound	Pose Ranking		$\Delta\Delta G_{xray \rightarrow flip}$	Program
	X-ray	Flip	(kcal/mol)	
1	1	2	1.0	Autodock Vina
2	-	1	-	Autodock Vina
3	1	2	1.6	Autodock Vina
4	3	-	-	Smina
5	-	4	-	Smina

Table S3. Docking results for compounds 1-3 in *Lm*NMT.

Compound	Pose Ranking		$\Delta\Delta G_{xray \rightarrow flip}$	Program
	X-ray	Flip	(kcal/mol)	
1	2	-	-	Autodock Vina
2	-	-	-	Autodock Vina
3	3	-	-	Autodock Vina

2.2 *Lm*NMT Compounds RMSD from Equilibrium MD Simulations.

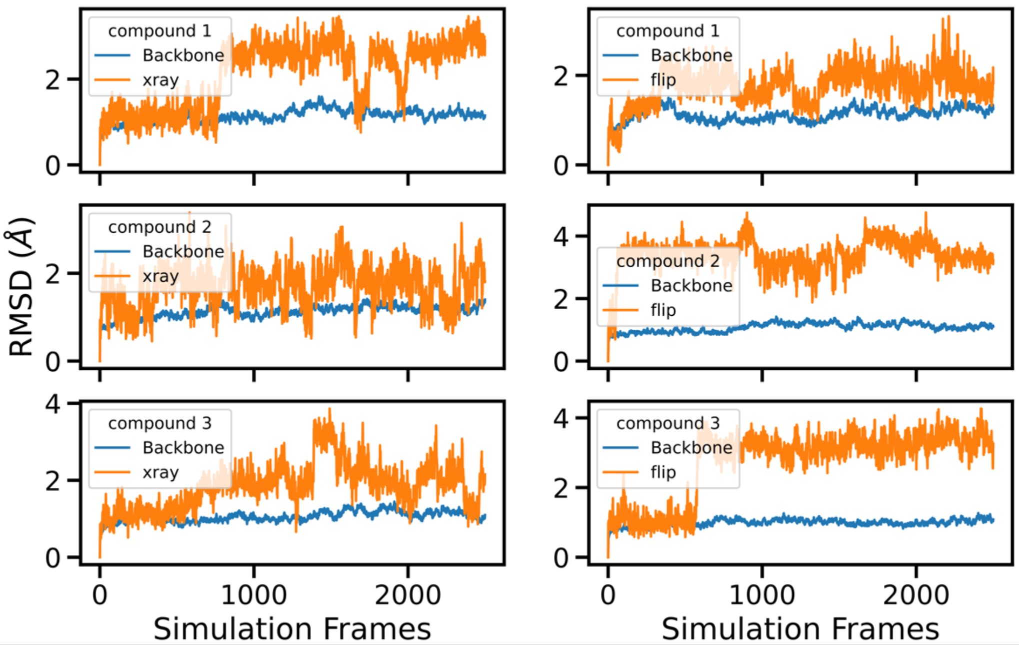

Figure S2. Protein backbone (CA, C, O, N) and ligand poses (all atoms) root mean square deviation (RMSD) for the *Lm*NMT system from equilibrium MD simulations using the CHARMM-CGenFF force field combination. RMSD analysis was done using MDAnalysis (final minimized structure used as reference); the protein backbone atoms were used for superimposition.

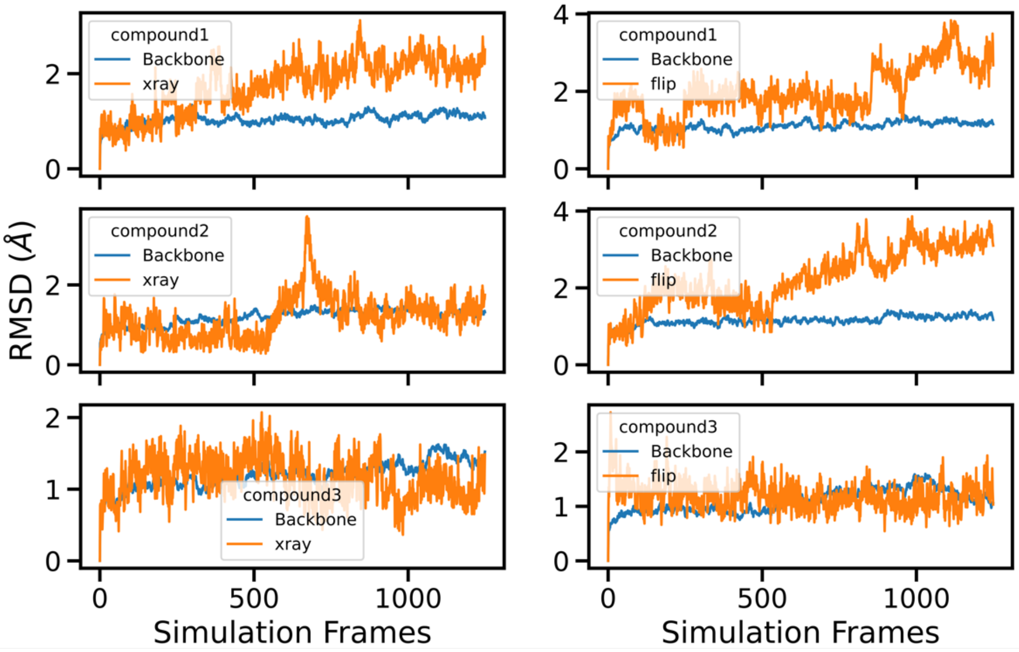

Figure S3. Protein backbone (CA, C, O, N) and ligand poses (all atoms) root mean square deviation (RMSD) for the *Lm*NMT system from equilibrium MD simulations using the OPLS-AA force field. RMSD analysis was done using MDAnalysis (final minimized structure used as reference); the protein backbone atoms were used for superimposition.

2.3 Multiple-distance restraints selected atoms

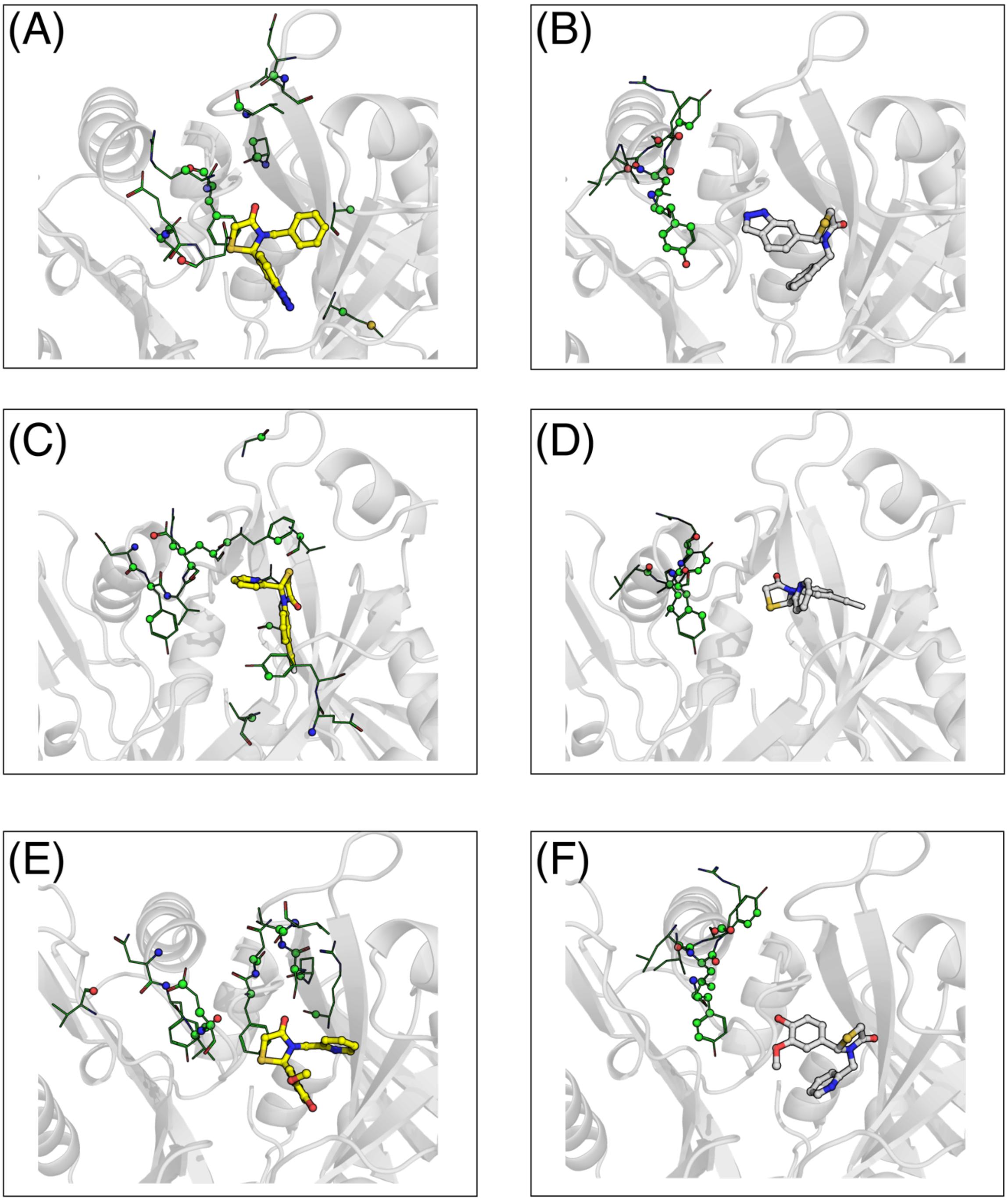

Figure S4. Protein atoms (shown as spheres) and ligand heavy atoms (shown as sticks) picked for multiple distance restraints (A,B: compound **1** X-ray and flipped poses respectively; C,D: compound **2** X-ray and flipped poses respectively; E,F: compound **3** X-ray and flipped poses respectively). These systems were simulated with the CHARMM-CGenFF force field.

Table S4. Inventory of protein and ligand atom pairs involved in multiple distance restraints.

**CHARMM-CGenFF**					
**1X-ray**	**1Flip**	**2X-ray**	**2Flip**	**3X-ray**	**3Flip**
PRO329CD:CAP	LEU75-O:CAD	ARG89CD:CAQ	LEU76O:CAH	PRO329CD:CAP	LEU76-O:CAE
PRO329CG:NAV	TYR73-O:CAC	ARG89CB:CAP	LEU76-C:N01	VAL81-CA:CAQ	TYR80-N:N01
ASN339-C:OAA	LEU76-O:CAE	GLN216N:CAO	ARG77-O:CAJ	VAL81-C:CAD	TYR73CD1:CAF
PRO329-N:CAR	LEU76CB:CAG	ARG327-C:CAJ	ARG77N:CAR	TYR80-N:NAV	TYR73CE1:CAC
MET377CB:CAE	TYR80-N:CAH	ARG89CA:CAG	TYR73CD1:CAK	GLU82-CD:CAL	LEU76-CB:CAG
ASP83-O:CAL	TYR80CA:CAP	TYR80CD1:CAC	LEU75-C:SAO	VAL332-N:CAU	LEU76-CA:CAI
ALA344CB:CAQ	LEU76CA:CAI	GLU82-CB:NAS	TYR73CE1:CAP	ASN79-N:CAK	TYR73-O:CAD
VAL332-C:SAO	LEU76CD2:CAQ	ASN79-N:CAR	TYR73-CA:OAA	GLU82-CA:CAF	LEU76-CG:CAQ
LEU340-N:CAK	TYR80-CB:CAJ	PHE88-C:CAE	LEU75-O:NAV	SER330-C:CAJ	LEU76CD2:CAJ
VAL332-N:CAU	ARG77-O:CAK	PHE88-CD2:CL	TYR80-N:CAS	THR331CA:CAS	TYR80-CA:CAP
PRO329-C:CAG	TYR80-CG:SAM	LEU399CB:OAA	TYR80-CA:CAT	GLU82CB:NAM	ARG77-C:CAK
ARG89-CB:CAJ	TYR80-CZ:CAN	GLY334-C:CAF	LEU76-CA:CAU	ARG327-C:OAB	ARG77-O:SAM
PHE90-N:CAS	TYR80CD2:OAA	LEU341CA:CAH	ARG77-CA:C02	GLU82-N:CAG	TYR80-CB:CAN
VAL81-C:CAB	TYR80CD1:NAS	VAL81-N:SAM	LEU76-CB:C01	ASN91-C:CAI	TYR80-CG:OAA
PHE90-CA:CAT	LEU76-CG:CAO	TYR80-CA:NAL	TYR80-CB:C03	VAL171-O:SAO	TYR80CD1:NAS
PHE90-C:NAM	TYR80CE1:CAR	GLU82OE1:CAI	TYR80CD2:CL1	ASN91-N:CAH	LEU76CD1:CAO
MET377SD:CAC	TYR80-OH:C01	GLU82-CA:CAN	TYR80-CG:C04	SER330CA:CAT	LEU76-C:CAR
PHE90-O:CAI	ILE72-O:C04	TYR217CE1:CAK	ARG77-CB:C05	ASN91-CA:CAR	LEU75-O:C02
PHE90-CB:CAH	TYR73-CA:N02	ASN79-C:CAD	LEU76-CG:C06	VAL81-O:CAE	LEU75-C:O02
GLU82-N:CAF	LEU76-N:C02			PHE90-CA:OAC	TYR80CE1:C03
PHE90-CG:NAN	TYR73CD1:CAF			THR331N:OAN	LEU76-N:C01
VAL81-CA:CAD	TYR73-CG:N01			THR331-C:CAA	ILE72-C:O01

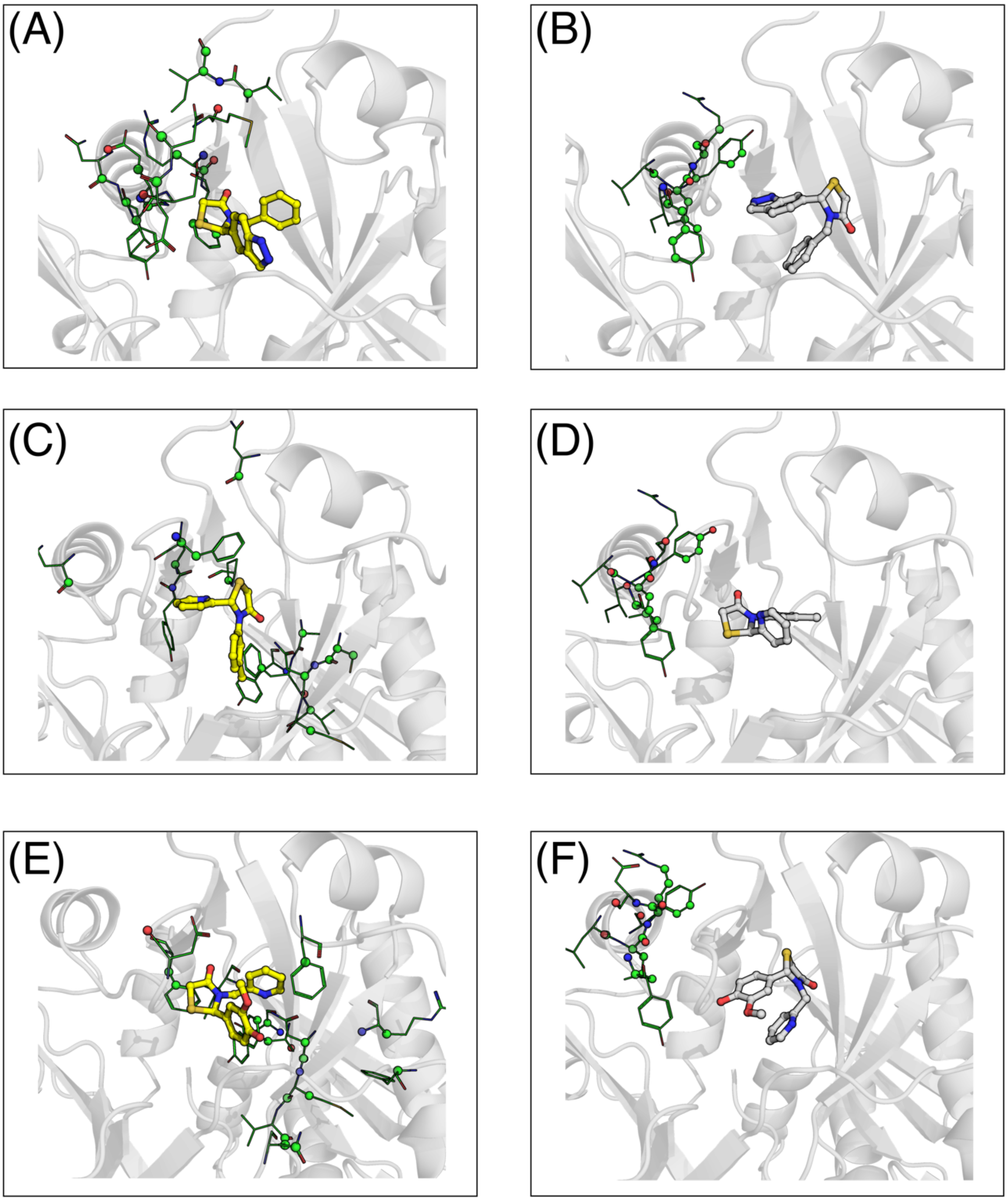

Figure S5. Protein (shown as spheres) and ligand heavy atoms (shown as sticks) picked for multiple distance restraints (A,B – compound 1 X-ray and flipped pose respectively; C,D – compound 2 X-ray and flipped pose respectively; E,F – compound 3 X-ray and flipped pose respectively). These systems were simulated with the OPLS-AA force field.

Table S5. Inventory of protein and ligand atom pairs involved in multiple distance restraints.

OPLS-AA					
**1X-ray**	**1Flip**	**2X-ray**	**2Flip**	**3X-ray**	**3Flip**
ASP83-O:CAL	LEU76-CA:CAD	ASN376-N:CAR	LEU76-O:CAH	ASN376-C:CAE	LEU76O:CAE
GLU82-CB:CAQ	LEU75-C:CAC	ALA344CA:CAO	LEU76-C:N01	PHE90-CG:OAN	ARG77O:N01
ASP84-C:CAP	LEU76-O:CAE	CYS375-CB:CAJ	ARG77-O:CAJ	MET377CB:NAM	TYR73CD1:CAF
GLU82OE1:NAV	LEU76-CB:CAG	CYS375-CA:CAP	TYR73-O:CAK	ASN376-O:CAD	TYR73-O:CAC
GLU82-N:CAD	TYR80-N:CAH	CYS375-C:CAH	TYR73CD1:CAP	MET377-N:CAG	LEU76-C:CAG
ASP85-C:OAA	LEU76-C:CAI	ASN376CA:CAQ	ILE72-O:OAA	TYR345-O:CAQ	LEU76-CA:CAI
VAL81-CA:CAF	ARG77-N:CAQ	PHE88-CA:CAG	LEU75-C:NAV	GLN216-CD:CAT	LEU76-CB:CAD
VAL81-C:CAB	ARG77-CA:CAJ	PHE88-CB:CL	TYR80-CA:CAS	TYR326-N:CAL	LEU76-CG:CAQ
PHE90CD2:NAN	ARG77-C:CAK	TYR326-N:NAS	TYR80-CB:CAT	ARG220-N:CAR	LEU76CD1:CAJ
PHE90-CG:CAH	ARG77-O:CAN	ASN376-O:CAK	LEU76-CA:CAU	TYR326-CA:NAV	TYR73CE1:CAK
ARG89-CB:CAE	TYR80-CB:OAA	TYR345CG:SAM	ARG77-N:CAR	ASP85-O:CAP	ARG77-N:CAN
ILE333-C:CAU	LEU76-CG:CAO	ASN79-C:CAD	LEU75-O:SAO	MET377-C:CAF	ARG77-CA:OAA
PHE90CD1:CAT	TYR80-CA:CAR	ASN91-CA:CAI	LEU76-CB:C02	GLN216-CB:CAJ	ARG77-C:CAO
ILE333-N:CAR	TYR80-CG:NAS	PHE88-N:CAE	TYR73CE1:C01	ARG220-CB:CAH	ARG77-CB:CAR
TYR80-C:CAC	ARG77CG:SAM	ASN339-C:CAF	LEU76CD1:C03	PHE90-CB:CAA	ARG77-CG:NAS
ILE333-CA:CAG	TYR80CD1:C01	PRO329CD:NAL	TYR80-CG:CL1	VAL378-C:CAI	ARG77CD:SAM
ASN79-C:CAK	TYR80CE1:CAP	VAL346CA:CAN	LEU76-CG:C04	TYR345CD1:CAK	ASP78-O:C02
VAL332CA:SAO	TYR73CD1:C04	TYR92-N:CAC	TYR73-CZ:C05	TYR345-CB:CAS	ASP78-N:O02
MET87-O:CAJ	LEU76CD1:C02	MET377CB:OAA	TYR73-OH:C06	ASN376-CA:OAB	TYR80-N:C03
ARG89-N:CAS	TYR73CE1:CAF			PHE392-CA:OAC	TYR80-CA:CAP
ARG89-O:NAM	ILE72-O:N01			ILE328-CB:CAU	LEU75-C:C01
ARG89-C:CAI	TYR73-CA:N02			PHE232-CG:SAO	LEU75-O:O01

2.4 ALF Convergence

2.4.1 HNE system

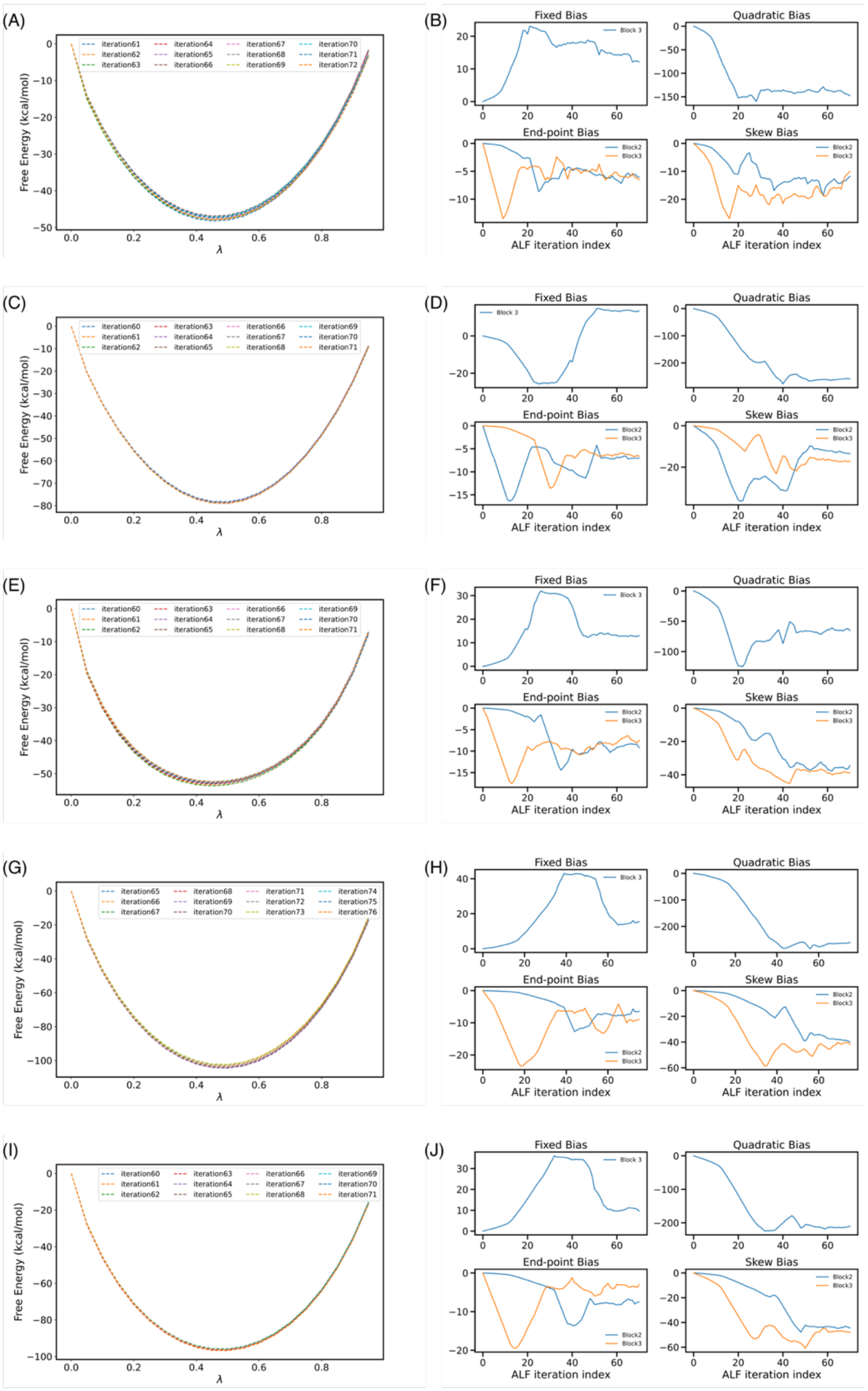

Figure S6. ALF predicted free energy landscape as a function of λ for HNE compounds **1-5** (A, C, E, G, I respectively; shown for the last 12 ALF iterations before the production run) using the CHARMM-CGenFF force field combination. The free energy landscape is generated using the ALF bias coefficients of the respective runs and using equation **S2.** Also, plotted together are the ALF bias coefficients (subplots B, D, F, H, J) as a function of MSλD runs. The ALF bias coefficients have the same dimension as energy and are expressed in kcal/mol.

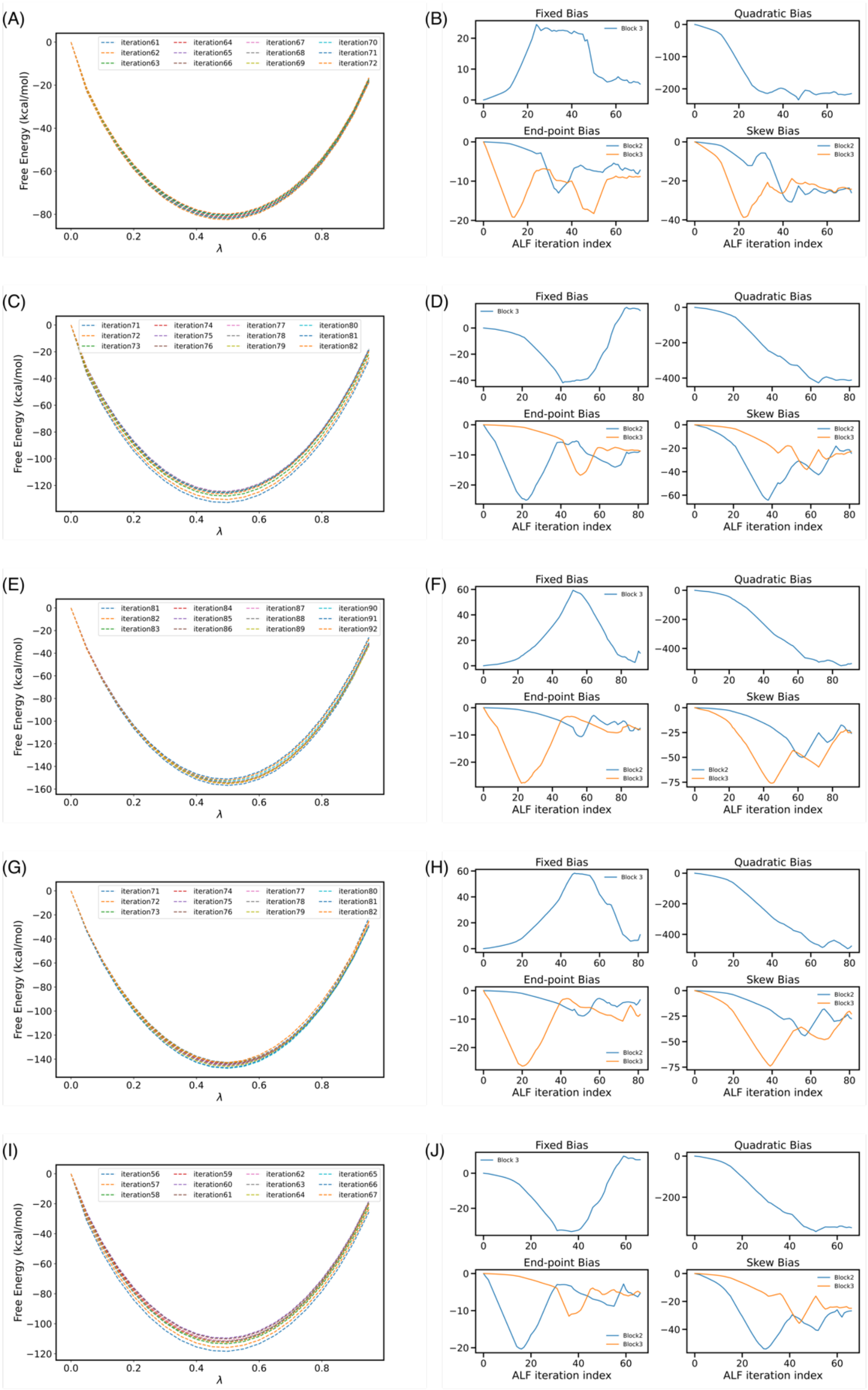

Figure S7. ALF predicted free energy landscape as a function of λ for HNE compounds **1-5** (A, C, E, G, I respectively; shown for the last 12 ALF iterations before the production run) using the OPLS-AA force field combination. The free energy landscape is generated using the ALF bias coefficients of the respective runs and using equation **S2.** Also, plotted together are the ALF biases coefficients (subplots B, D, F, H, J) as a function of MSλD runs. The ALF bias coefficients have the same dimension as energy and are expressed in kcal/mol.

Table S6. Total number of transitions between the ligand poses and the fraction physical ligand (FPL)[9] for the human neutrophil elastase (HNE) system. The number of transitions is the total number of transitions per ns from 5 replicas of 30 ns each and the FPL is the average over 5 replicas of 30 ns each.

Compound	CHARMM-CGenFF		OPLS-AA	
	Transitions	FPL	Transitions	FPL
1	194	0.385	226	0.527
2	432	0.514	188	0.546
3	254	0.6	173	0.558
4	246	0.318	169	0.575
5	221	0.598	201	0.302

2.4.2 *Lm*NMT

Table S7. Total number of transitions between the ligand poses and the fraction physical ligand (FPL). The number of transitions is the total number of transitions per ns from 5 replicas of 30 ns each and the FPL is the average over 5 replicas of 30 ns each.

Compound	CHARMM-CGenFF		OPLS-AA	
	Transitions	FPL	Transitions	FPL
1	153	0.611	178	0.642
2	189	0.517	165	0.437
3	216	0.483	177	0.761

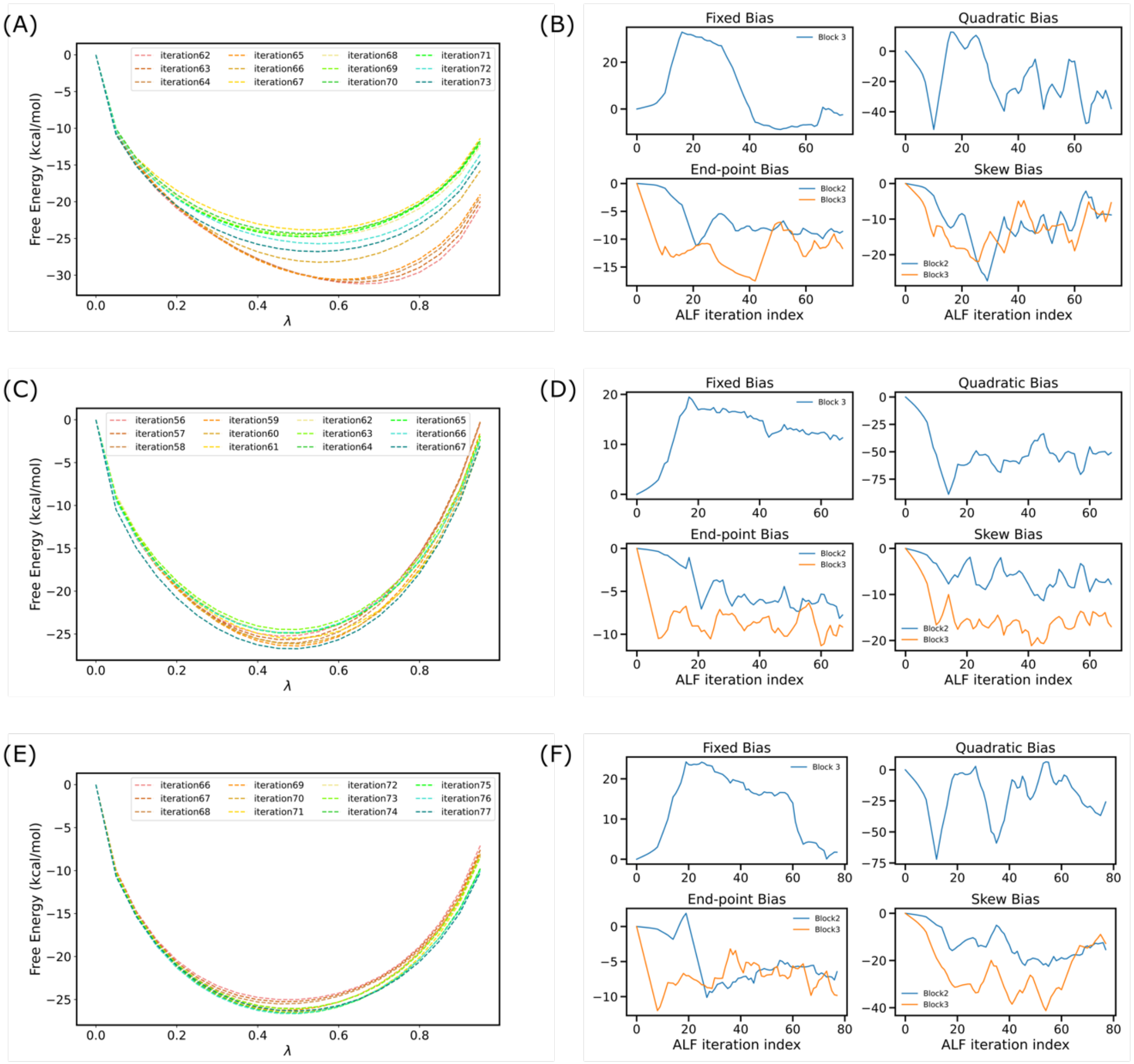

Figure S8. ALF predicted free energy landscape as a function of λ for *Lm*NMT compounds **1-3** (A, C, E respectively; shown for the last 12 ALF iterations up to the production run) using the CHARMM-CGenFF force field combination. The free energy landscape is generated using the ALF bias coefficients of the respective runs and using equation **S2.** Also, plotted together are the ALF biases coefficients (subplots B, D, F) as a function of MSλD runs. The ALF bias coefficients have the same dimension as energy and are expressed in kcal/mol.

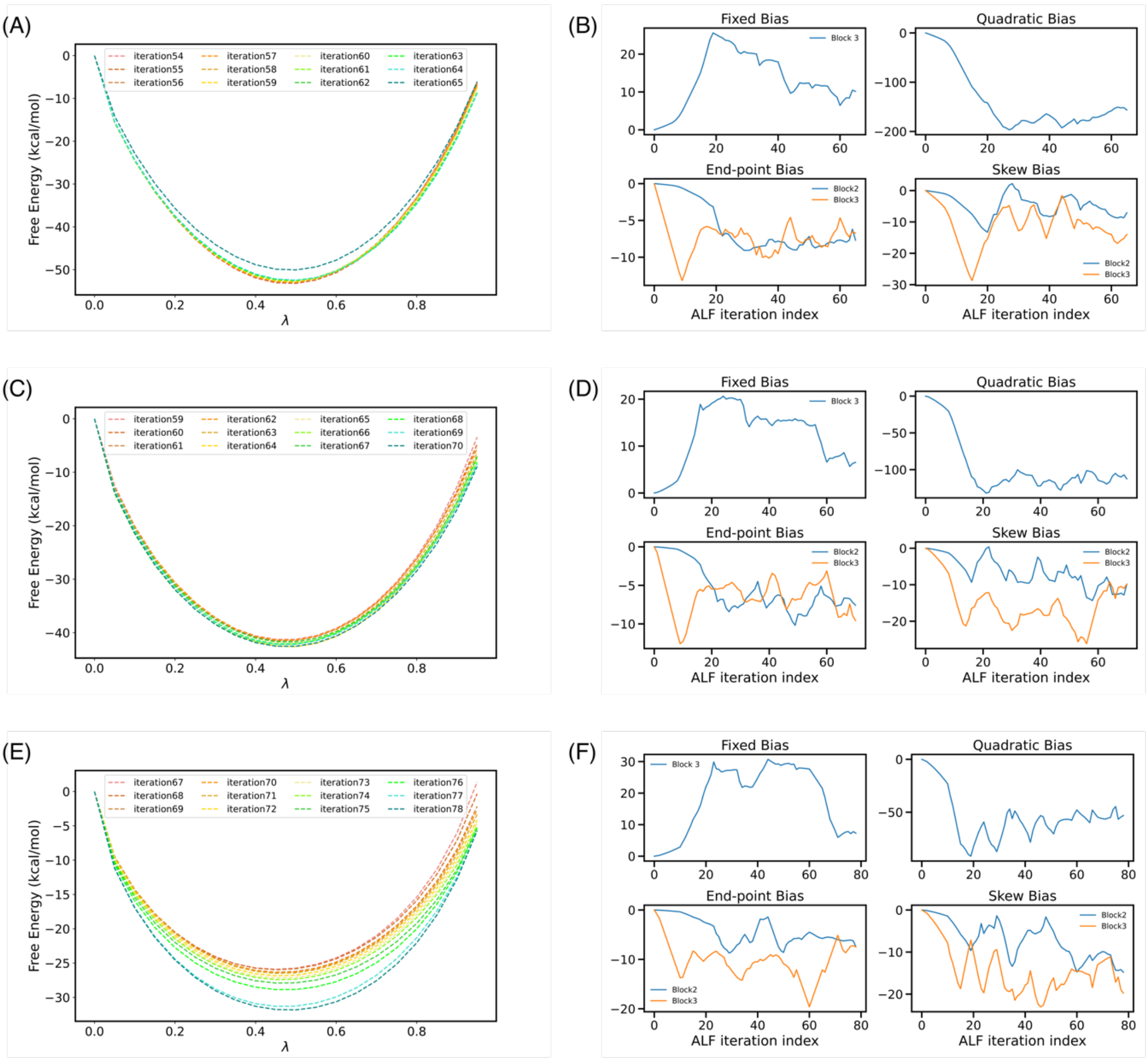

Figure S9. ALF predicted free energy landscape as a function of λ for *Lm*NMT compounds **1-3** (A, C, E respectively; shown for the last 12 ALF iterations up to the production run) using the OPLS-AA force field. The free energy landscape is generated using the ALF bias coefficients of the respective runs and using equation **S2.** Also, plotted together are the ALF biases coefficients (subplots B, D, F) as a function of MSλD runs. The ALF bias coefficients have the same dimension as energy and are expressed in kcal/mol.

2.5 MSλD Predicted Relative Binding Affinities for the HNE Compounds

Table S8. Relative binding free energies predicted for HNE compounds using the OPLS-AA force field with the X-ray pose. The term $\Delta\Delta G_{bind}^{xray}$ (equivalent to $\Delta\Delta G_{MS\lambda D}^{FF}$) is calculated as described in our previous work.[10]

Compound	$\Delta\Delta G_{bind}^{xray}$ (kcal/mol)	
	e14fac = 0.5	e14fac = 1.0
1	0.0 ± 0.5	0.0 ± 0.2
2	-5.2 ± 0.6	-2.7 ± 0.6
3	-2.4 ± 0.4	-0.5 ± 0.7
4	-3.9 ± 0.1	-1.2 ± 0.3
5	-1.5 ± 0.4	1.2 ± 0.2

Table S9. Comparison of MSλD predicted absolute binding free energies for HNE compounds obtained using the OPLS-AA force field against experimental activity data reported by Nussbaum et al.[11,12] ($\Delta G_{expt} = RTln(IC_{50})$)[a] for the X-ray poses. The term ΔG_{bind}^{xray} (equivalent to $\Delta G_{MS\lambda D}^{FF}$) is calculated as described in our previous work.[10]

Compound (X-ray pose)	IC_{50} (nM)	ΔG_{expt} (kcal/mol)	ΔG_{bind}^{xray} (kcal/mol) e14fac = 0.5	ΔG_{bind}^{xray} (kcal/mol) e14fac = 1.0
1	6.5	-11.1	-9.0 ± 0.5	-11.4 ± 0.2
2	0.17	-13.3	-14.2 ± 0.6	-13.8 ± 0.6
3	28	-10.3	-11.2 ± 0.4	-10.9 ± 0.7
4	0.54	-12.6	-14.4 ± 0.1	-13.2 ± 0.3
5	3.5	-11.5	-10.1 ± 0.4	-9.4 ± 0.2
RMSE		-	1.5	1.0
Pearson's R		-	0.77	0.76
Spearman's ρ		-	0.6	0.7

[a]Since the measurements were carried out at substrate concentrations significantly below K_m so, $K_i \approx IC_{50}$ in accordance with the Cheng-Prusoff equation.

Table S10. Predicted absolute binding free energies for the flipped poses of HNE compounds.

**Compound**	ΔG_{expt}	ΔG_{bind}^{xray}	$\Delta\Delta G_{xray \rightarrow flip}$	ΔG_{bind}^{flip}
		**(kcal/mol)**		
1	-11.1	-9.0 ± 0.5	3.3 ± 0.1	-5.7 ± 0.5
2	-13.3	-14.2 ± 0.6	13.2 ± 0.2	-1.0 ± 0.6
3	-10.3	-11.2 ± 0.4	8.9 ± 0.1	-2.3 ± 0.4
4	-12.6	-14.4 ± 0.1	12.8 ± 0.2	-1.6 ± 0.2
5	-11.5	-10.1 ± 0.4	7.7 ± 0.3	-2.4 ± 0.5

[a] $\Delta G_{bind}^{flip} = \Delta\Delta G_{xray \rightarrow flip} + \Delta G_{bind}^{xray}$

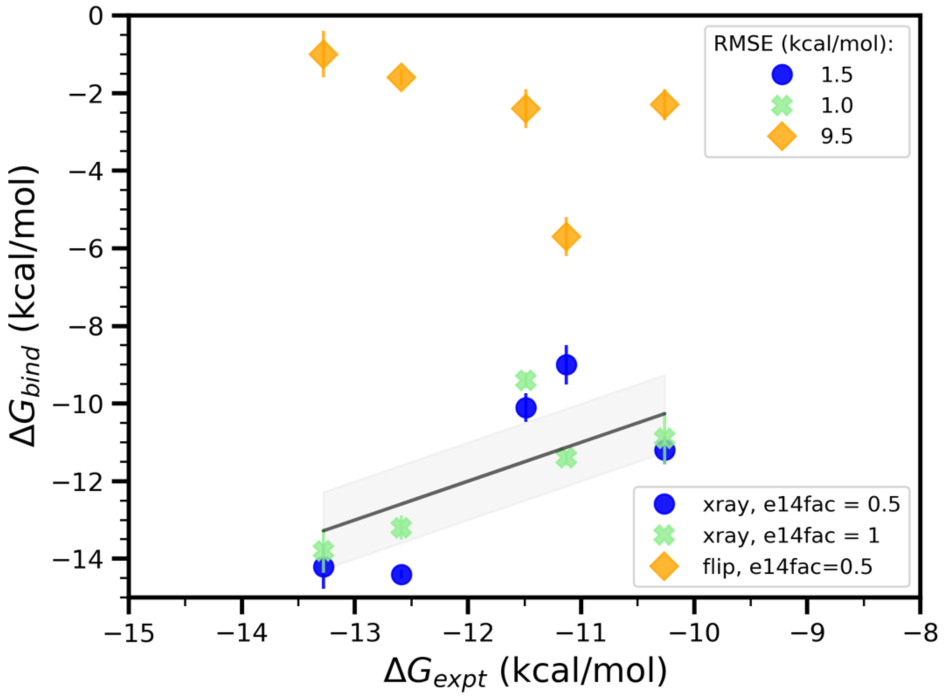

Figure S10. Comparison of predicted and experimental absolute binding free energies of HNE compounds using either the X-ray pose (blue dots) for all the five ligands or using the flipped poses (orange diamonds) for all the five ligands. The predicted absolute binding affinities using the flipped poses correlate poorly against the experimental data (Pearson's correlation coefficient, R = -0.5) whereas the predicted absolute binding affinities using the X-ray poses are in excellent agreement with experimental data (R = 0.77 and 0.76; **Table S9**). Using an e14fac value of 1 (ie no scaling of the coulomb term

between atoms interacting through three bonds) instead of 0.5 leads to an improvement of the RMSE from 1.5 to 1 kcal/mol.

2.6 Analysis of MSλD Trajectories

Table S11. List of polar interactions from λ-dynamics simulations. The heavy atoms (O, N) of the ligand (highlighted in red) that are within 3.5 Å from the protein residue heavy atoms (O, N) are shown here.

Compound[a]		CHARMM-CGenFF	OPLS
1X-ray		O-OG_SER330	--
1Flip	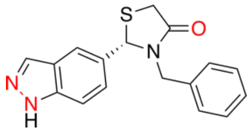	O-ND2_ASN376, N-N_PHE90, N-OG_SER330, N-OG_SER330	O–ND2_ASN376, N-N_PHE90
2X-ray		O-ND2_ASN376	O-ND2_ASN376
2Flip	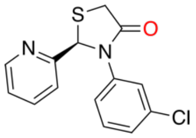	O-OG_SER330	O-N_PHE90
3X-ray	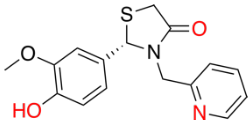	O-OG_SER330	N-ND2_ASN376, O-OG_SER330, O-N_ASP396
3Flip		O-ND2_ASN376, O-N_PHE90	O-ND2_ASN376

[a] compounds structures are shown with stereochemistry corresponding to the X-ray pose.

Table S12. Average number of hydrogen bonds per frame from λ-dynamics trajectories. Only ligand dominant frames ($\lambda > 0.99$) were used. Hydrogen bond criteria (D-H--A distance ≤ 2.4 Å and D-H--A angle ≥ 130 where, D = donor atom, H = hydrogen atom bonded to the donor atom, A = acceptor atom). Prot-water-lig stands for water-bridged protein-ligand hydrogen bonds.

Compound	CHARMM-CGenFF		OPLS	
	Prot-lig[a]	Prot-water-lig[b]	Prot-lig[a]	Prot-water-lig[b]
1X-ray	2.0	0.5	0.0	0
1Flip	1.6	0.2	1.0	0
2X-ray	0.4	1.3	0.7	0
2Flip	1.0	0.0	0.1	0
3X-ray	1.2	1.4	1.3	0.1

3Flip	0.7	1.6	0.9	0

[a] protein-ligand hydrogen bonds, [b] water bridged hydrogen bonds between protein and ligand

2.7 FEP/MBAR to Evaluate Contributions of Protein-Ligand Multiple Distance Restraints

Table S13. Free Energy of removing restraints from the X-ray and flipped poses using a combination of MBAR and Zwanzig's perturbation formula. MBAR is used until the penultimate window. The unidirectional forward free energy change from penultimate to final window is calculated using Zwanzig's perturbation formula (FEP).

Compound 2 (*Lm*NMT; CHARMM36m/CGenFF)	ΔG ($kcal/mol$)		$\Delta G^{FEP/MBAR}_{pose^{**} \rightarrow pose}$
**Pose**	**MBAR**	**FEP**	**(kcal/mol)**
X-ray	-7.3 ± 0.0	-3.2	-10.5 ± 0.0
Flip	-11.7 ± 0.1	-5.8	-17.5 ± 0.1

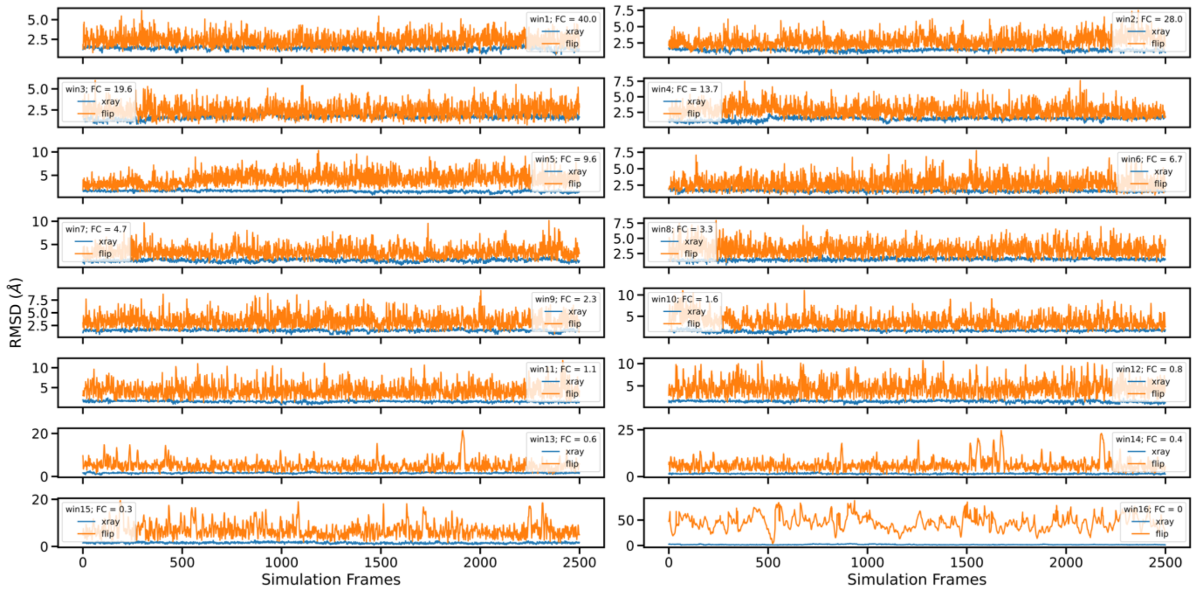

Figure S11. Compound **2** (*Lm*NMT; CHARMM36m/CGenFF), RMSD for the X-ray and flipped poses in the FEP windows for removing restraints from the X-ray pose.

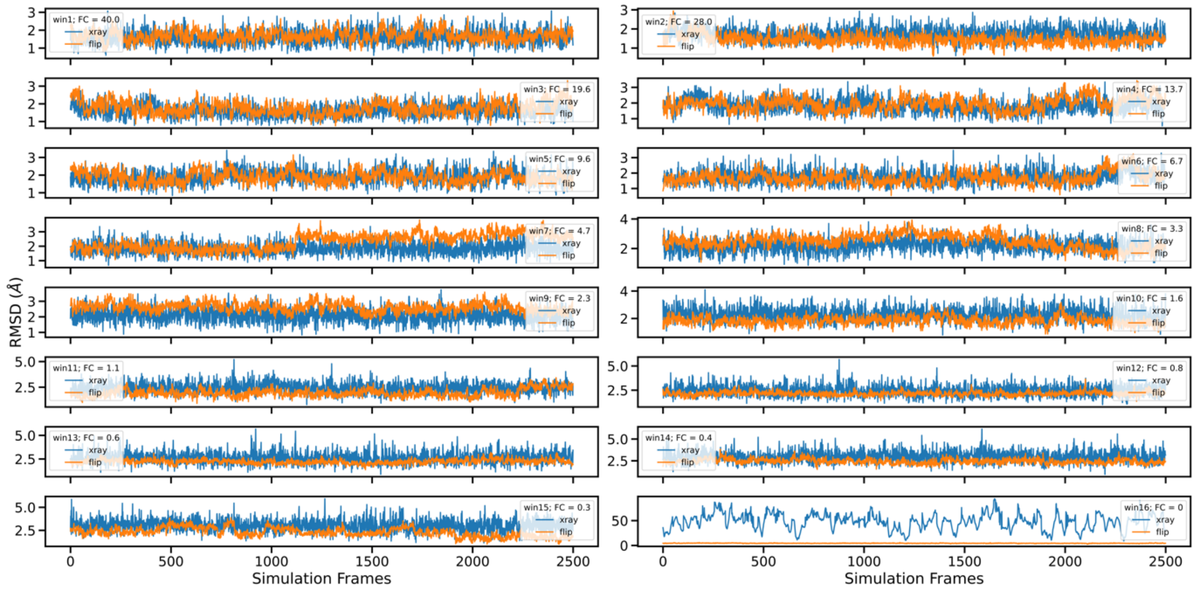

Figure S12. Compound **2** (*Lm*NMT; CHARMM36m/CGenFF), RMSD for the X-ray and flipped poses in the FEP windows for removing restraints from the flipped pose.

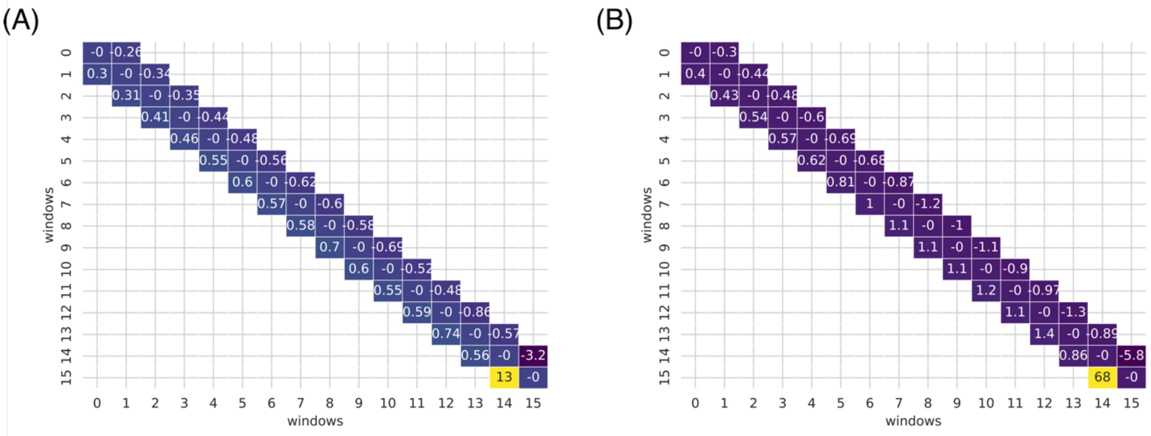

Figure S13 Free energy differences of removing the protein-ligand multiple distance restraints between the neighboring windows in both forward and reverse direction using Zwanzig's perturbation formula. (a) X-ray pose, (b) flipped pose. There is very poor phase space overlap between the last window (index 15) and the second last window (index 14). This poor phase space overlap can be attributed to the high heavy-atom RMSD of the substituent with λ=0 and NOE force constant = 0.

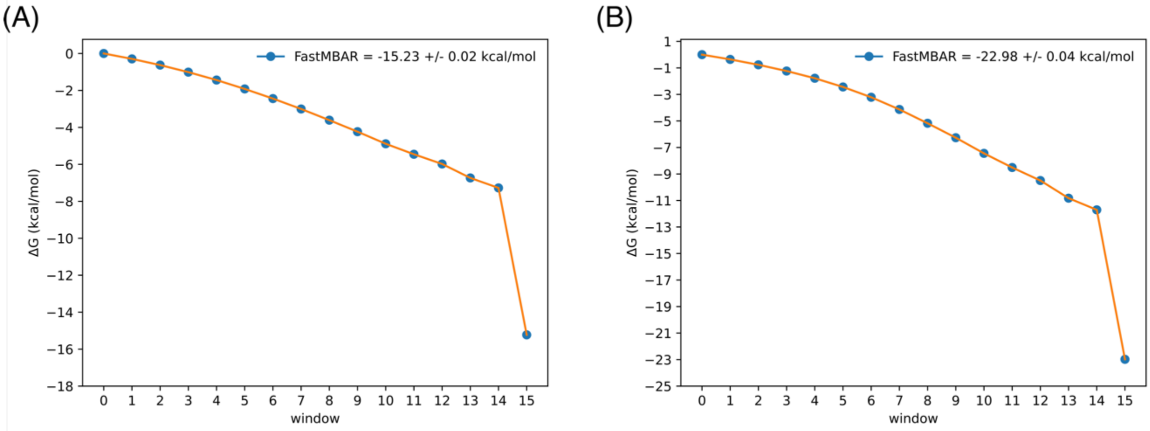

Figure S14. FastMBAR PMF plot (a) removing restraints from the X-ray pose. (b) removing restraints from the flipped pose.

2.8 *Lm*NMT Compounds: Convergence of Free Energies from One-Step Perturbation Simulations

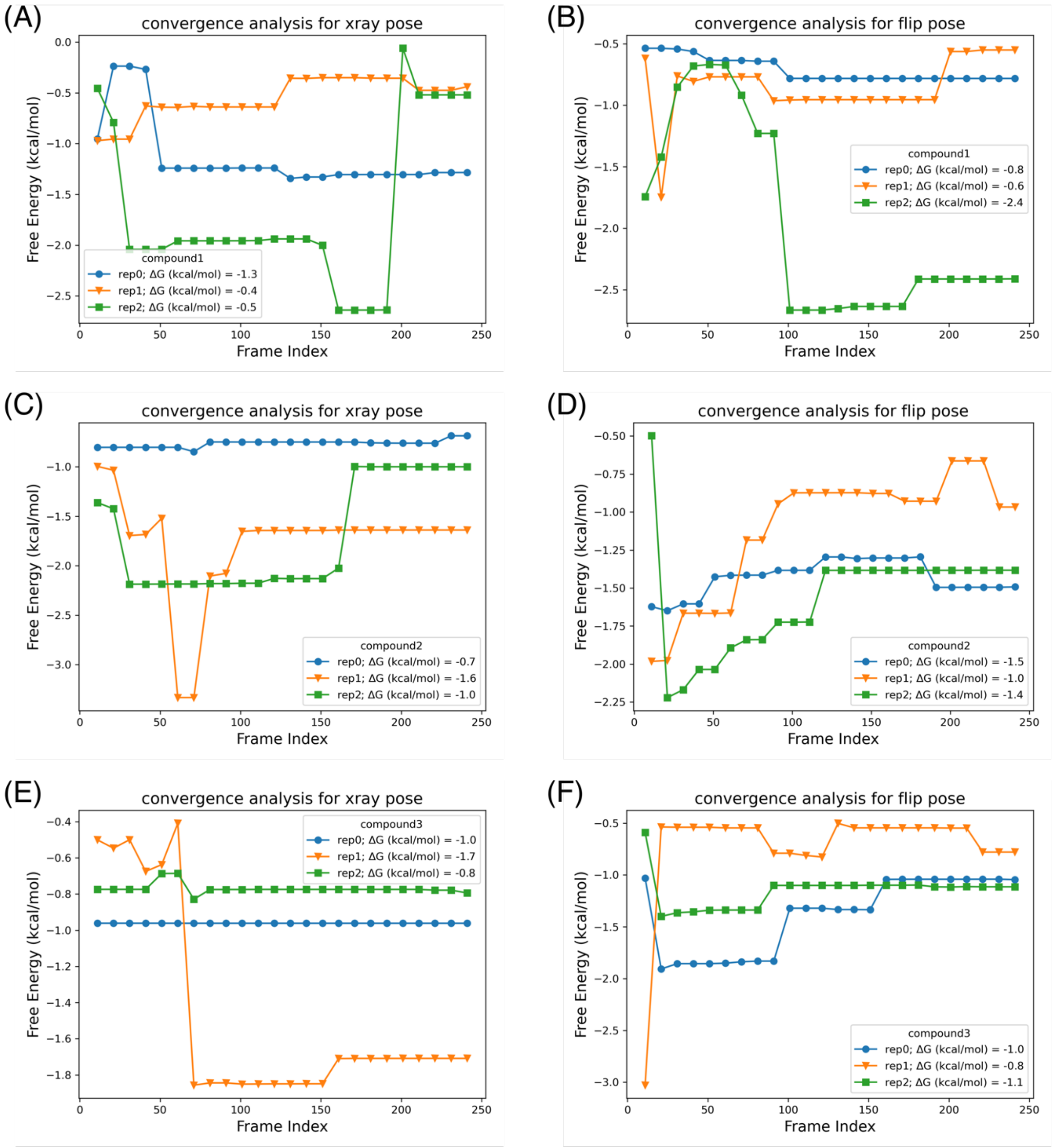

Figure S15. Free energies corresponding to the cost of removing multiple distance restraints calculated using Zwanzig's perturbation formula as a function of number of simulation frames. These simulations were run with the CHARMM36m-CGenFF force field combination in three replicates.

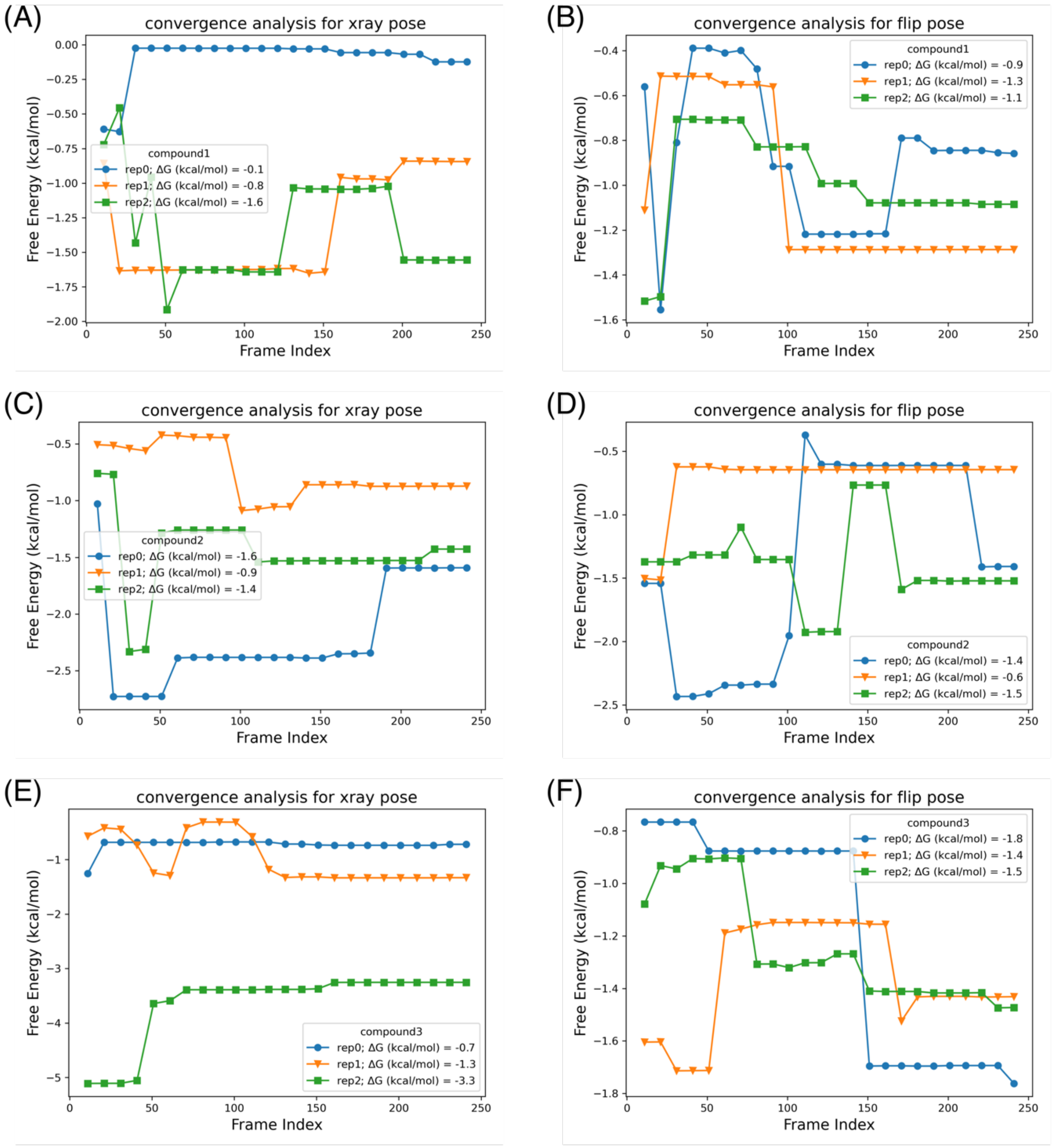

Figure S16. Free energies corresponding to the cost of removing multiple distance restraints calculated using Zwanzig's perturbation formula as a function of simulation time. These simulations were run with the OPLS-AA force field combination in three replicates.

2.9 *Lm*NMT Compounds: RMSF from One-Step Perturbation Simulations

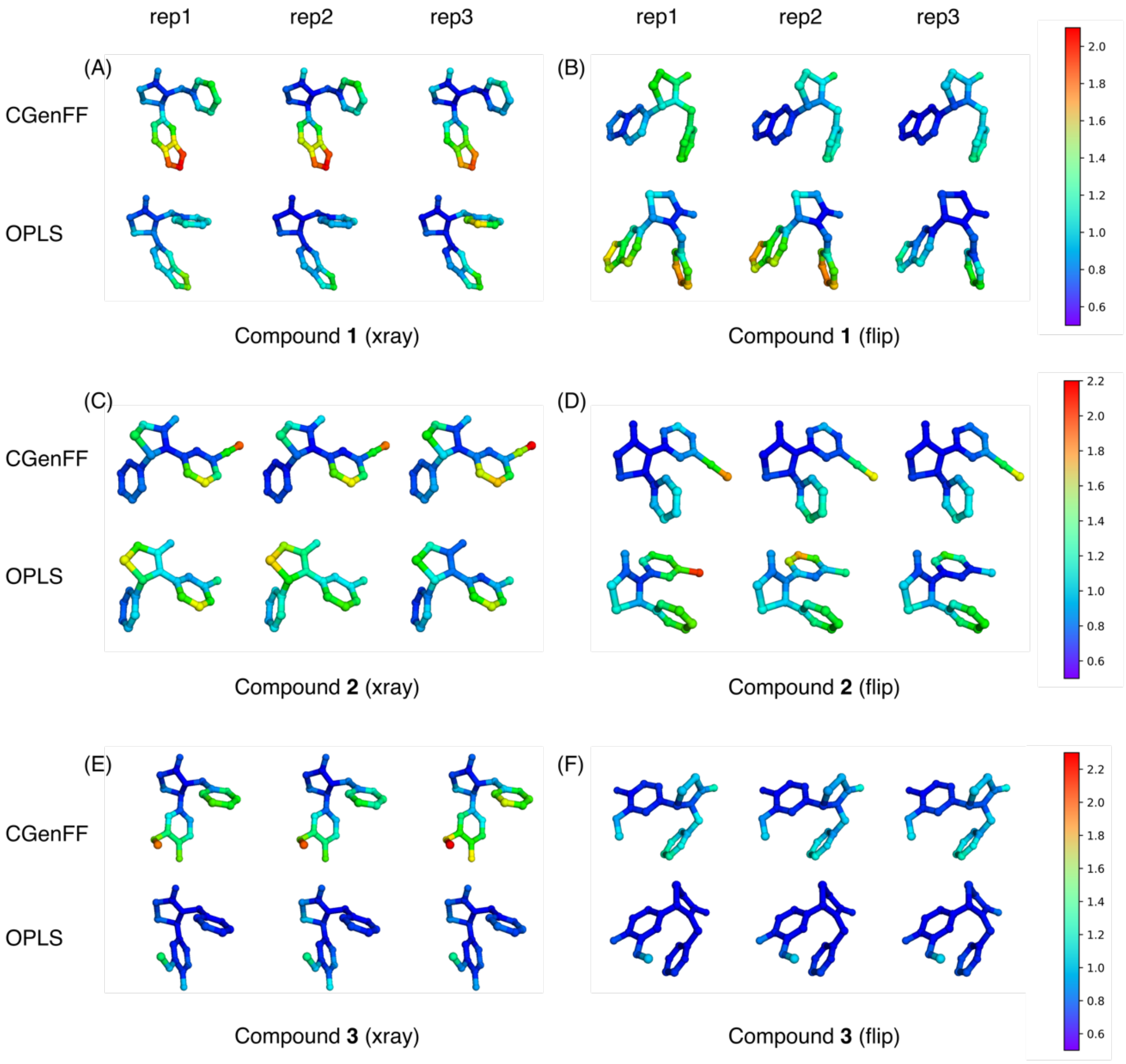

Figure S17. Root mean square fluctuations of the compound poses from three independent replicas of one-step perturbation simulations. Both the ligand poses were kept at $\lambda = 0.5$ whereas the force constant for the multiple distance restraints remain unchanged. In these simulations, dihedral angles were scaled by λ. The figure was prepared with PyMOL.

3. Cumulative Simulation Times

Table S14. Cumulative simulation time spent on the ALF flattening and production λ-dynamics simulations for HNE.

System	Compound	Number of atoms	Simulation time (ns)			
			ALF Flattening	Equilibration	Production	Total
HNE (CHARMM)	1	28330	25	25	300[a]	350
	2	28400	25	25	150	200
	3	28389	15	25	150	190
	4	28410	30	25	150	205
	5	28418	25	25	150	200
HNE (OPLS)	1	28330	25	25	150	200
	2	28400	35	25	150	210
	3	28389	25	75[a]	150	250
	4	28410	26	25	150	201
	5	28418	20	25	150	195

[a] The equilibration or production simulations were run more than once which is reflected in the total simulation time spent.

Table S15. Cumulative simulation time spent on the ALF flattening and production MSλD simulations for HNE ligands (**1-5**).

Side	e14fac	Number of atoms	Simulation time (ns)			
			ALF Flattening	Equilibration	Production	Total
Water	0.5	4997	30	25	125	180
Complex	0.5	38202	13	25	200	238
Water	1.0	4997	55	25	125	205
Complex	1.0	38202	13	25	200	238

Table S16. Cumulative simulation time spent on the ALF flattening and production λ-dynamics simulations for *Lm*NMT.

System	Compound	Number of atoms	Simulation time (ns)			
			ALF Flattening	Equilibration	Production	Total
*Lm*NMT (CHARMM)	1	76395	16	25	350[a]	391
	2	76383	11	25	150	186
	3	76400	21	25	150	196
*Lm*NMT (OPLS)	1	76738	18	25	150	193
	2	76448	11	25	650[a]	686
	3	76356	21	50[a]	150	196

[a] These equilibration or production simulations were run more than once which is reflected in the total simulation time spent.

References

(1) Vanommeslaeghe, K.; MacKerell, A. D. Automation of the CHARMM General Force Field (CGenFF) I: Bond Perception and Atom Typing. *J. Chem. Inf. Model.* **2012**, *52*, 3144–3154. DOI: 10.1021/ci300363c.

(2) Vanommeslaeghe, K.; Raman, E. P.; MacKerell, A. D. Automation of the CHARMM General Force Field (CGenFF) II: Assignment of Bonded Parameters and Partial Atomic Charges. *J. Chem. Inf. Model.* **2012**, *52*, 3155–3168. DOI: 10.1021/ci3003649.

(3) Dodda, L. S.; Cabeza de Vaca, I.; Tirado-Rives, J.; Jorgensen, W. L. LigParGen Web Server: An Automatic OPLS-AA Parameter Generator for Organic Ligands. *Nucleic Acids Res.* **2017**, *45*, W331–W336. DOI: 10.1093/nar/gkx312.

(4) Trott, O.; Olson, A. J. AutoDock Vina: Improving the Speed and Accuracy of Docking with a New Scoring Function, Efficient Optimization, and Multithreading. *J. Comput. Chem.* **2009**, NA-NA. DOI: 10.1002/jcc.21334.

(5) Koes, D. R.; Baumgartner, M. P.; Camacho, C. J. Lessons Learned in Empirical Scoring with Smina from the CSAR 2011 Benchmarking Exercise. *J. Chem. Inf. Model.* **2013**, *53*, 1893–1904. DOI: 10.1021/ci300604z.

(6) Ding, X.; Vilseck, J. Z.; Brooks, C. L., III. Fast Solver for Large Scale Multistate Bennett Acceptance Ratio Equations. *J. Chem. Theory Comput.* **2019**, *15*, 799–802. DOI: 10.1021/acs.jctc.8b01010.

(7) Gilson, M. K.; Given, J. A.; Bush, B. L.; McCammon, J. A. The Statistical-Thermodynamic Basis for Computation of Binding Affinities: A Critical Review. *Biophys. J.* **1997**, *72*, 1047–1069. DOI: 10.1016/S0006-3495(97)78756-3.

(8) Michel, J.; Verdonk, M. L.; Essex, J. W. Protein−Ligand Complexes: Computation of the Relative Free Energy of Different Scaffolds and Binding Modes. *J. Chem. Theory Comput.* **2007**, *3*, 1645–1655. DOI: 10.1021/ct700081t.

(9) Hayes, R. L.; Vilseck, J. Z.; Brooks, C. L., III. Addressing Intersite Coupling Unlocks Large Combinatorial Chemical Spaces for Alchemical Free Energy Methods. *J. Chem. Theory Comput.* **2022**, *18*, 2114–2123. DOI: 10.1021/acs.jctc.1c00948.

(10) Gartan, P.; Khorsand, F.; Mizar, P.; Vahokovski, J. I.; Cervantes, L. F.; Haug, B. E.; Brenk, R.; Brooks, C. L., III; Reuter, N. Investigating Polypharmacology through Targeting Known Human Neutrophil Elastase Inhibitors to Proteinase 3. *J. Chem. Inf. Model.* **2024**, acs.jcim.3c01949. DOI: 10.1021/acs.jcim.3c01949.

(11) von Nussbaum, F.; Li, V. M.-J.; Allerheiligen, S.; Anlauf, S.; Bärfacker, L.; Bechem, M.; Delbeck, M.; Fitzgerald, M. F.; Gerisch, M.; Gielen-Haertwig, H.; et al. Freezing the Bioactive Conformation to Boost Potency: The Identification of BAY 85-8501, a Selective and Potent Inhibitor of Human Neutrophil Elastase for Pulmonary Diseases. *ChemMedChem* **2015**, *10*, 1163–1173. DOI: 10.1002/cmdc.201500131.

(12) von Nussbaum, F.; Li, V. M.; Meibom, D.; Anlauf, S.; Bechem, M.; Delbeck, M.; Gerisch, M.; Harrenga, A.; Karthaus, D.; Lang, D.; et al. Potent and Selective Human Neutrophil Elastase Inhibitors with Novel Equatorial Ring Topology: In Vivo Efficacy of the Polar Pyrimidopyridazine BAY-8040 in a Pulmonary Arterial Hypertension Rat Model. *ChemMedChem* **2016**, *11*, 199–206. DOI: 10.1002/cmdc.201500269.
